# Intracellular Bacteria in Plants: Elucidation of Abundant and Diverse Cytoplasmic Bacteria in Healthy Plant Cells Using In Vitro Cell and Callus Cultures

**DOI:** 10.3390/microorganisms9020269

**Published:** 2021-01-28

**Authors:** Pious Thomas, Christopher M. M. Franco

**Affiliations:** 1 Thomas Biotech & Cytobacts Centre for Biosciences, Amruthahalli, Bengaluru 560092, India; 2 Department of Medical Biotechnology, College of Medicine & Public Health, Flinders University, Bedford Park, Adelaide, SA 5042, Australia

**Keywords:** 16S rRNA gene taxonomic profiling, confocal laser scanning microscopy, endophytic bacterial microbiome, fluorescent in situ hybridization (FISH), metagenomics, next-generation deep sequencing, plant-microbe interactions, plant tissue culture, *Vitis vinifera* L.

## Abstract

This study was initiated to assess whether the supposedly axenic plant cell cultures harbored any cultivation-recalcitrant endophytic bacteria (CREB). Adopting live-cell imaging with bright-field, fluorescent and confocal microscopy and bacterial 16S-rRNA gene taxonomic profiling, we report the cytoplasmic association of abundant and diverse CREBs in long-term actively maintained callus and cell suspension cultures of different plant species. Preliminary bright-field live-cell imaging on grape cell cultures showed abundant intracellular motile micro-particles resembling bacteria, which proved uncultivable on enriched media. Bacterial probing employing DNA stains, transmission electron microscopy, and Eubacterial FISH indicated abundant and diverse cytoplasmic bacteria. Observations on long-term maintained/freshly established callus stocks of different plant species—grapevine, barley, tobacco, *Arabidopsis*, and medicinal species—indicated intracellular bacteria as a common phenomenon apparently originating from field shoot tissues.Cultivation-independent 16S rRNA gene V3/V3–V4 amplicon profiling on 40-year-old grape cell/callus tissues revealed a high bacterial diversity (>250 genera), predominantly Proteobacteria, succeeded by Firmicutes, Actinobacteria, Bacteriodetes, Planctomycetes, and 20 other phyla, including several candidate phyla. PICRUSt analysis revealed diverse functional roles for the bacterial microbiome, majorly metabolic pathways. Thus, we unearth the widespread association of cultivation-recalcitrant intracellular bacteria “Cytobacts” inhabiting healthy plant cells, sharing a dynamic mutualistic association with cell hosts.

## 1. Introduction

Plants are known to host a diverse array of microorganisms, including bacteria, fungi, and archaea, collectively called “plant microbiome”, of which the specialized organisms that colonize the tissues internally without adverse effects on the host are generally described as endophytes [1,2,3]. Earlier cultivation-based studies indicated that endophytic bacteria were present in low numbers, and were perceived majorly as root colonizers across different plant species [1,2,3]. Microscopically, they proved largely inter-cellular inhabitants after the initial invasion through root cortex, and migrating to shoot tissues through vascular and apoplastic channels [2,4,5]. Initial cultivation-independent studies indicated the prevalence of normally uncultivable endophytic bacteria in the root tissues of some field plants [6,7]. Next-generation sequencing (NGS) and metagenomics-based cultivation-independent studies subsequently revealed high taxonomic and functional diversity of endophytic bacteria as root colonizers [8,9] and subsequently in the shoot tissues in uncultivable or cultivation-recalcitrant form [10,11]. Considerable information has been emerging on endophytic bacterial colonization and distribution inside plants, their transmission including seed/vertical transmittance and the beneficial effects [3,12,13,14,15,16].

Plant tissue cultures, generally initiated from the shoot or other tissues after surface sterilization, are generally considered aseptic and axenic [17,18]. Researchers and commercial propagators often proceed with the visibly clean plant tissue cultures, assuming freedom from microorganisms. Contrary to this, micropropagated stocks were demonstrated to harbor bacteria in a covert or non-obvious form, detected through culture-indexing, where the easily cultivable microorganisms grow on enriched bacteriological media [19,20]. Further, it was documented that diverse bacteria enter in vitro from field tissues and appear as contaminants, or survive in visibly clean cultures in a subdued or uncultivable form [20,21,22]. While endophytic bacteria are considered mostly inter-cellular or apoplastic colonizers [1,2,5], occasional reports cited them as intracellular colonizers [23,24], including the cytoplasmic bacteria “Cytobacts” documented with some plant systems [25,26].

Microbial association with plant tissue cultures has been a common issue centered on micropropagation and in vitro conservation, with several publications highlighting contamination management and culture cleansing [19,20,27,28]. Plant cell and callus cultures are used extensively for basic investigations in plant biology, elucidating metabolic pathways, and the biopharming of novel biomolecules [29,30]. Various applications with plant cell and callus cultures embark on the assumption of their axenic status and the attribution of their properties solely to the host plant cells. On the other hand, recent NGS-based studies on different plant species have indicated the prevalence of a high diversity of cultivation-recalcitrant endophytic bacteria in field shoots, their in vitro entry through surface-sterilized tissues, and the unexpected sustenance in healthy micropropagated cultures in uncultivable form [26,31] including freshly initiated healthy callus stocks [22]. We have maintained the cell suspension and callus cultures of different plant species for extended periods ranging from a few years to over four decades for use in basic and applied research [32,33]. This study was initiated with the primary objective of assessing if such long-term, actively maintained healthy cultures harbored any endophytic bacteria. The present study unearths the “Cytobacts”, comprising abundant and diverse cultivation-recalcitrant endophytic bacteria (CREB) as integral associates of healthy plant cells in vitro with the origin ascribable to the field source tissues.

## 2. Material and Methods

### 2.1. Plant Tissue Cultures

The cell/callus cultures used in this study included 15 stocks of *Vitis vinfera*, three stocks *of Hordeum vulgare*, and 10 stocks of different medicinal species, *Catharanthus roseus*, *Ajuga reptans*, *Fallopia sachalensis*, *F. japonica*, *Polygonum cuspidatum* (Table S1). These cultures were initiated from shoot tissues of field-grown Australian plants (leaf, stem, nodes, petiole, berry) or were obtained from researchers in different countries and cultured in vitro for 10 to >40 years with regular sub-culturing to fresh medium at monthly interval. Grape cv. “Gamay Fréaux” cell suspension stock “FU01” initiated from “FC01” callus stock [33,34] and sub-cultured fortnightly at Flinders University, Adelaide, Australia, formed the primary candidate for detailed experiments. Culture medium “GC02” comprising Gamborg’s B5 constituents, 0.2 mg L^−1^ each kinetin and NAA, as per [32], was used for culture maintenance. Cell suspension cultures in stationary phase (2–3 w) and callus stocks 4–8 w after sub-culturing were generally employed in different studies.

Additionally, fresh callus cultures of grapevine, *Arabidopsis*, and tobacco were originated in Bengaluru, India, from surface-sterilized tender shoot and petiole tissues of field/glasshouse grown plants. Grapevine “Flame Seedless” and “Thompson Seedless” cultures were initiated and maintained as described elsewhere [22]. *Arabidopsis* and tobacco callus stocks were originated from tender stem/petiole tissues of the growth chamber or glasshouse-grown seedlings. Tissues were surface sterilized using NaOCl (2% available chlorine) for 3–6 min and cultured on “GC02” callusing medium [32] under dark incubation. 

### 2.2. Cultivation Based Testing of Plant Tissue Cultures for Bacterial Association

All the above-mentioned cultures displayed active growth without any visible indications of microbial presence. They were further tested for any non-obvious or covert bacterial association as per the three-step screening procedure [19]. This included diligent visual examination and indexing of medium and tissue by transferring these components to nutrient agar (NA) at 37 °C and tryptone soy agar (TSA) at 30 °C and observing for any bacterial colony growth for 2–7 days. Additionally, ten other bacteriological media/incubating conditions (Table S1) were employed to check for the presence of conventionally cultivable bacteria for up to one month.

### 2.3. Protoplast Preparation from Cell Cultures

This was done to separate the cells from the cluster to ascertain the intracellular colonization by endophytic bacteria employing callus stocks of grape “FC01”, *Arabidopsis* and tobacco. The callus stocks were treated with 0.2 µm filter-sterilized mixture of 0.5% cellulase, 0.25% driselase, and 400 mM mannitol, prepared in MS basal medium [35] without sugar. The cells were separated with gentle pipetting up and down the solution with a vide mouth Pasteur pipette. The solution was removed after 2–4 h by slow spinning (2000× *g*) for 2 min. 

### 2.4. Bright-Field Microscopy on Cell, Callus, and Protoplasts

Fresh cells from suspension and callus cultures were examined in filter-sterilized (0.2 μm) distilled water post-autoclaving (FDW) or autoclaved MS medium under 100× oil objective directly or after applying the bacterial stain, Gram’s crystal violet or safranin (0.1–0.5%). An Olympus BX50 photomicroscope equipped with QImaging Micropublisher RTV 5.0 megapixel digital camera (Olympus, Tokyo) and a Leica DM 2000 optical microscope with a DFC-295 digital live camera and Leica Application Suite (LAS) software version 3.8 (Leica Microsystems CMS GmbH, Wetzlar, Germany), were employed for bright-field and phase-contrast microscopy. Live movie files were recorded under bright-field for 20–30 s using the LAS software. The DM2000 microscope allowed generous horizontal scanning and 30–40 µm vertical scanning. Adobe Photoshop (7.0) was used for editing the images, and Microsoft Windows 10.0 Movie Maker for video editing. 

### 2.5. Epi-Fluorescence Microscopy

DAPI (10 µg mL^−1^) and Hoechst 33342 (4–20 µM), in sterile water, were employed for staining bacteria and plant cell nuclei, and fluorescein di-acetate (FDA) 20 µg mL^−1^ in phosphate-buffered saline (PBS) for assessing plant cell viability. LIVE/DEAD BacLight^®^ bacterial viability kit L13152 (Molecular Probes, Thermo Fisher Scientific) containing SYTO-9 and propidium iodide (PI) was employed for detecting live/dead bacteria with the stocks prepared in FDW (https://assets.thermofisher.com/TFS-Assets/LSG/manuals/mp07007.pdf). For staining tissue-associated bacteria, 10 µL of 2× SYTO-9 and 1× PI, prepared as per the kit instructions, were applied onto FDW-washed plant cells (final concentrations of 12 and 30 µM, respectively) on clean slides and observed after 10 min under 40× objective of the Olympus AX70 model epi-fluorescence microscope, fitted with the excitation/dichroic mirror/emission filters 340–380/400/435–485 for DAPI, 490/505/515–545 for SYTO-9, FDA and 6-carboxy-fluorescein (FAM), and 515–550/565/575–615 for PI. Images were captured using the Leica DMLS 16-bit monochrome camera and AnalySIS software. Images were colored and further processed with Adobe Photoshop 7.0 (Adobe Systems). Adjustments to the entire images in brightness or color balance were made, in some instances, for improved bacterial detection, the extent of which is reflected in the color shade of the scale bar. Staining was also tried, employing the kit constituents prepared in PBS, PO_4_ buffer (0.5 M; pH 7.4), or MS medium and with or without washing off the stain. Cells were also used for staining after fixing in 2% formalin or paraformaldehyde, or formalin–glacial acetic acid–ethanol (1:1:18).

### 2.6. Confocal Laser Scanning Microscopy (CSLM)

Leica TCS SP5 Spectral confocal system (Leica Microsystems, Wetzlar, Germany) with DM 1650B inverted microscope equipped with long pass filter cube “A” for blue emission, “I3” for green emission and “N2.1” for red emission, and the lasers 405 nm Diode, Argon, DPSS 561 and HeNe 633, were used for examining the plant cells stained with various fluorophores. Tissue samples were examined using the 63× HCX PL APO 1.2 water objective, excited with 405 nm Diode for DAPI, and SYTO-9 and PI with Argon lasers 488 and 561, respectively (set at 50%), and collected at 408–480, 495–560, 570–700 nm, respectively. Images were acquired at 1-airy using LAS AF1.6.1 software (Leica Microsystems) in 512 × 512 pixel format, at 400 Hz, and with a frame averaging of 3–4. 

Further, an LSM 5 LIVE confocal laser scanning microscope (CLSM), equipped with a 488 nm laser and supported by LSM software (Carl Zeiss Inc., Jena GmbH, Germany), was used for microscopic examination of SYTO-9 stained cell and callus stocks, as described elsewhere [36]. Image J was used to generate .avi files from time-lapses and z-stacks. Since these did not provide a realistic time-scale video, the direct recording was done from the LSM monitor using a Sony digital camera.

### 2.7. Electron Microscopy

For Transmission electron microscopy (TEM), grape cells were fixed in 2.5% glutaraldehyde in 0.1 M sodium cacodylate buffer (pH 6.4) for 2 h at 25–26 °C, washed thrice with buffer and post-fixed overnight in 1% osmium tetroxide in buffer. Cells were buffer-washed, dehydrated (20, 50, 70, 90 and 100% ethanol) and washed thrice in absolute ethanol. Samples were infiltrated and embedded in Spurr resin. Sections (approx. 70 nm) were cut (Reichert OMU2 microtome) using a diamond knife (Electron Microscopy Sciences, Hatfield, PA, USA) and dried on 200 hex TEM grids (Gilder Grids, Grantham, UK) overnight on a plate warmer. After double staining in 2% uranyl acetate (25 min) and 0.5% lead citrate (10 min) with a distilled water washing in between, they were imaged in a Tecnai 12 Biotwin TEM (FEI Company, Hillsboro, OR, USA) employing Gatan 791 multiscan camera (Gatan, Pleasanton, CA, USA).

### 2.8. Distinguishing Intracellular Bacteria from Plant Micro-Organelles

With a view to ascertaining if the DNA probes SYTO-9 and DAPI cross-stained plant mitochondria, the staining of fresh cells of grape callus/cell suspensions with the MitoTracker^®^ Orange CMTMRos (MTO) kit (Cat. No. M7510; Thermo Fisher Scientific, Waltham, MA, USA) was tried. The stock (1 mM) was prepared in DMSO as per the kit instructions, and dilutions of 25–500 nM in sterile water were tried for tissue staining, applying the 25 µL probe solution over the cells on a glass slide (4–5 min), followed by rinsing with water. Cells were imaged mounted in water using a Leica DM-LB2 epi-fluorescence microscope (Leica Microsystems, Wetzlar, Germany) with UV, N2.1, GFP, and I3 filters, and under bright-field using the DF320 color camera and LAS software (version 3.8). The tissue homogenate from grape “FC01” callus was observed directly or in the presence of safranin (0.01%) under bright-field and phase-contrast using the DM2000 microscope to get a clearer picture of mitochondria/plastids versus bacterial cells.

Further, with a view to check if the smaller intracellular motile particles corresponded to peroxisomes that move along actin filaments, grape and tobacco callus cultures were treated with actin disrupting 2, 3, butanedione monoxime (15, 30 or 60 mM for up to 2 h) or latrunculin B (1 µM) [37,38]. Additionally, microtubule-disrupting nocodozol (3.3 µM) was used to see the effect on intracellular particle motility. 

### 2.9. Fluorescent In Situ Hybridization (FISH)

FISH [39] was undertaken using 2–3 w old “FU01” grape stock, employing 5′-FAM labeled probes Eub338 (5′GCTGCCTCCCGTAGGAGT-3) [40], targeting most bacteria, EubII+III (5′GCWGCCACCCGTAGGTGT for bacterial groups not covered by Eub338 [41], LGC354ab (5′YSGAAGATTCCCTACTGC for all Firmicutes [42], or 5′-Cy3 labeled HCG69a (5′TATAGTTACCACCGCCGT) targeting Actinobacteria [39], ALF969 (5′TGGTAAGGTTCTGCGCGT) for *α*-Proteobacteria, BET42a (5′GCCTTCCCACTTCGTTT) for *β*-Proteobacteria and GAM42a (5′GCCTTCCCACATCGTTT) for *γ*-Proteobacteria [39]. Eub338ns (5′ CGACGGAGGGCATCCTCA) and HCG69/ns (5′ATATCAATGGTGGCGGCA) were employed as non-sense negative controls for FAM and Cy3 labels, respectively. The probes were prepared at GeneWorks, Adelaide, Australia.

The working protocol evolved after a series of trials involved the pre-treatment of grape cells using cellulase Y-C (0.5%) for 30 min in MS medium with 3% sucrose (pH 6.0) and 0.01% TritonX-100, followed by washing with hybridization buffer (900 mM NaCl, 20 mM Tris HCl pH 7.4, 5 mM EDTA pH 8.0; no detergents) by slow spinning at 500 rpm in a table-top centrifuge (5 min; 3×). Hybridization was carried out in 500 µL microfuge tubes using 100 µL permeabilized cell aliquots and single probes (200 ng) at 46 °C for 3–4 h in a water-bath under dark conditions. Washing was undertaken using the buffer as above with 0.01% TritonX-100 at 48 °C with three solution changes at 15 min. The cells were finally dispersed in hybridization buffer and examined after mounting in carbonate buffered glycerol (1:2) under 100× oil objective of AX70 fluorescent microscope. Sample, similarly treated but without the label, was employed to check the auto-fluorescence of plant cells. Non-specific binding, as observed with non-sense probe controls, could be eliminated by incubating the washed tissue samples overnight in wash buffer (400 µL) containing 0.001% TritonX-100 at 4 °C. The choice, concentration, and duration of exposure to detergents (SDS, TritonX-100) during pre-treatments, hybridization and washing steps appeared critical in improving the permeability of the plant as well as bacterial cells in fresh grape tissue without imparting significant adverse effects on the former. Cells were also tried in FISH with Eub338, as above, after fixation in 2% formalin/paraformaldehyde, or formalin–glacial acetic acid–ethanol fixative.

### 2.10. 16S rRNA Gene V3 Taxonomic Profiling on Grape Cell Culture

Grape “Gamay Fréaux” cell culture “FU01” was taken up for 16S rRNA gene V3 region-based taxonomic profiling to get a cultivation-independent estimate of bacterial diversity. About 10 g tissues from two independent stock cultures were homogenized in FDW (0.1 *w*/*v*) and DNA was extracted as per PowerFood^®^ microbial DNA isolation kit (MO BIO Laboratories Inc., Carlsbad, CA, USA) standard protocol. After checking for the bacterial 16S rRNA gene with PCR amplification using 27F and 1492R-Y primers [21], DNA samples from two independent batches (referred to as MG3-1 and MG3-2) were submitted to M/s Scigenom Labs Pvt Ltd., Cochin, India (www.scigenom.com) for eubacterial 16S rRNA V3 hyper-variable region profiling, which was essentially carried out as per Illumina protocol Part #15044223 Rev. B (http://web.uri.edu/gsc/files/16s-metagenomic-library-prep-guide-15044223-b.pdf) as described elsewhere [11]. 

The bioinformatics analyses involved fastaq quality checking, read filtering steps, chimera screening (UCHIME in USEARCH tool), and merging the paired-end sequences to single-end reads, keeping a minimum Phred score of 30 and 10 bp overlap. Taxonomic assignments to Operational Taxonomic Units (OTUs) were made through the bioinformatics pipeline QIIME (Quantitative Insights Into Microbial Ecology; http://qiime.org/) [43] and employing Greengenes as the 16S rRNA reference database for taxonomic assignment as described elsewhere [11]. Final OTU assignments were made after singleton removal (<2), keeping the OTU similarity threshold at 0.97 and taxonomy identification confidence of 0.80 through the de novo approach. Alpha-diversity and Beta-diversity were assessed with the circular graphical representation of reads using Krona.

### 2.11. 16S rRNA Gene V3–V4 Taxonomic Profiling on Grape Callus Tissue

DNA was extracted from the grape “FC01” callus using a new lot of PowerFood® kit as per the standard protocol. A second sample was processed with an extended lysis step (10 min at 70 °C) before the bead-beating step, as per the kit protocol. The two DNA samples (referred to as MG11 and MG12, respectively) were submitted to M/s Xcelris Labs Ltd., Ahmadabad, India (www.xcelrislabs.com) for 16S rRNA V3–V4 hyper-variable region profiling targeting Eubacteria and Archaea including data processing and two rounds of QIIME analysis to exclude the reads corresponding to chloroplast and mitochondria, undertaken as described elsewhere [11]. Further, a comparative assessment of the phylogenetic taxa in MG12 in relation to the more recently initiated callus stocks of grapevine cultivars “Flame Seedless” and “Thompson Seedless” (MG18 and MG19, respectively, as per [22]) were made through VENN analysis.

### 2.12. Bacterial Activation and Identification

Based on the information on the association of diverse bacteria with the grape cell culture/callus stocks, the possibility of activating non-cultivable bacteria to cultivation was tried with the use of host tissue homogenate (HTH) under low nutrition [44]. Grape cells “FU01” two weeks after the previous sub-culturing were washed off the medium to remove all external organisms and homogenized in a mortar in PBS (100 mg mL^−1^). After the settling of the debris, 100 µL of HTH was mixed with equal volume of nutrient broth and 800 µL FDW in 1.5 mL tubes employing five replications. The samples were spotted and dilution-plated on NA and TSA soon after, followed by spotting the suspension (2 µL × 25 lots) at 1–2 day intervals for a fortnight. The colony growth that emerged after 4–7 days of stationary incubation of the sample was used to isolate constituent organisms that were identified through 16S rRNA sequence analysis [44].

### 2.13. Functional Profiling of Bacterial Communities in Grape Callus

The PICRUSt v1.1.1 (phylogenetic investigation of communities by reconstruction of unobserved states) tool [45] was used to predict the functional composition of the metagenome using marker gene data and a database of the reference genome. The reads of MG11 and MG12 samples that were assigned as chloroplast, mitochondria and no blast hit were removed. A total of 2076 stitched reads that were obtained after quality check with QIIME v1.8.0 were used to create a closed reference OTU table with a Greengenes core set reference database. The PICRUSt tool was used to predict functional composition based on KEGG pathways, Clusters of Orthologs Groups (COG) classification, and RFam, as described [26].

### 2.14. Microscopic Observations on Field Plant Tissues

Bright-field microscopy was employed to assess the intracellular bacterial presence in field plant tissues of the major experimental species covered in this study. Tender shoots from pruned canes of grapevine “Flame Seedless” and “Thomson Seedless”, the hypocotyls part of 1-week-old seedlings of *Arabidopsis* and petiole sections from glasshouse grown periwinkle and tobacco, were employed for this. These tissue sections were prepared from surface-sterilized tissues, as per [25], and video-graphed under bright field microscopy using the Leica DM 2000 optical microscope, as described, for cell cultures.

### 2.15. Accession Numbers

The 16S rRNA sequence data of bacteria activated in this study have been deposited with the NCBI Genbank with the accession numbers KC787046–KC787049. The 16S rRNA metabiome data have been deposited with NCBI with the Bioproject ID: PRJNA286272 (Submission IDs SUB974641 and SUB975951 for V3–V4 profiling, and SUB1729428 for V3 profiling).

## 3. Results

### 3.1. Callus and Cell Cultures

The cultures used in this study were cultured in vitro for 10 to >40 years, except for the new callus stocks of grapevine, *Arabidopsis*, and tobacco (five stocks) that were originated relatively more recently (Table S1). The results presented here are based primarily on grapevine “Gamay Fréaux” cell suspension culture “FU01” actively maintained for over three decades with fortnightly sub-culturing to the fresh culture medium, or “FC01” callus stock with monthly sub-culturing. The cells, tissue homogenate, or the growth medium from these cultures did not show any microbial colony growths during their indexing/testing, employing ten different bacteriological media at 27 and 37 °C, indicating the absence of cultivable bacterial association (Table S1). Mere reference to cells in this report pertains to the host/plant cells.

### 3.2. Live Imaging of Grape Cells under Bright-Field Microscopy

Bright-field microscopy on live-cell cultures under high magnification (1000×) was the primary route that revealed the intracellular bacterial associations. The non-fixed grape suspension cultures comprised cells of 20–40 µm in small clusters with red-pigmented patches at 100× (Figure S1). Plastids (≥5 µm) and mitochondria (≥2 µm) became apparent at 400× magnification with some intra-tissue micro-particle motility. Under 100× oil immersion objective, varying amounts of motile micro-particles became obvious, aided with low safranin usage (0.005%), which primarily appeared as “Brownian motion” (Movie S1). The mobile units included small, medium or large cocci and fine rods displaying slow motion or active motility, and the latter indicated it was not a mere random event. Careful examination of single cells in FDW indicated that the particles were mostly intracellular (Movie S2), including the vacuolar particles observed in callus cells (Movie S3). The similarity of these motile particles to bacteria which were activated to cultivation from the “FU01” stock (e.g., *Staphylococcus pasteuri*, *Bradyrhizobium elkanii*), or that were isolated as endophytes from other plant sources, suggested these as endophytic bacteria (Movie S4). Micro-videography was essential to depict the motile units, which was not clear with still images. All cells in a cluster, particularly those with high organelle inclusions, did not display active micro-particles but were seen frequently in older cultures and peripheral cells in a cluster that could be better focused. Motility could be induced within static cells by exerting gentle pressure over the cover-glass, indicating that cytoskeleton interruption perhaps helped release them. Variations in size and motility patterns of such micro-particles were observed from cell to cell in different clusters. In some instances, plastids and mitochondria showed displacement in the bacterial current, possibly after the cytoskeleton disruption. Very few bacterial cells were observed in the culture medium relative to the host cells.

Cell cultures fixed in formalin (2–4%; 1–4 h) often showed a loss in particle motility with abundant static grainy particles corresponding to bacteria (Figure S1), while the motile activity continued unhindered in some instances. Tissue staining with crystal violet or safranin (0.5–0.05%) rendered most motile particles non-mobile, while some persevered with safranin. Bacterial motility was unaffected with low safranin (0.001–0.005%), but in formalin-fixed cells, safranin rendered them low- or non-motile, revealing abundant cellular bacteria. The above observations also held good for pure cultures of different bacteria examined under live microscopy or subjected to various fixatives and stains. 

### 3.3. Live imaging of Grape Cells under Epi-Fluorescence Microscopy

**Figure 1:**
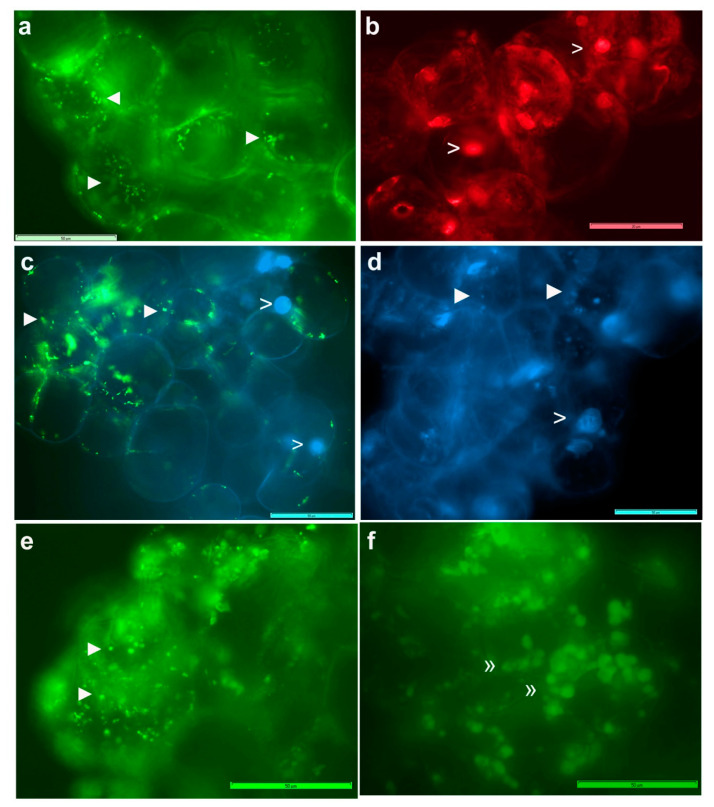
Grape cells stained with SYTO-9 and propidium iodide (PI) from Live/Dead bacterial staining kit, or with DAPI with or without cell permeabilization (40× objective). (**a**) Grape cells (FU01) show green fluorescing bacteria along the cell periphery and in the cytoplasm with SYTO-9, (**b**) cell nucleus stained with PI, (**c**) combined staining with SYTO-9 and DAPI showing nucleus with DAPI and fluorescing blocks of bacteria with SYTO-9, (**d**) extended staining with DAPI alone for 60 min, showing some cytoplasmic bacteria, (**e**) staining with SYTO-9 after pre-treating with Triton X-100 in water at 0.01% for 30 min showing cytoplasmic bacteria, (**f**) pre-treating with Triton X-100 0.1% in PBS for 1 h displaying cell organelles matching the expected size of mitochondria and plastids. (Symbols ►, > and » indicate bacterial cells, nuclei and organelles, respectively.) Adjustments to the entire images in brightness or color balance have been made to improve the bacterial detection extent of which is reflected in the color shade of the scale bar (bar = 50 µm).

Cell staining using FDA indicated >95% cell viability for grape “FU01” suspension cultures of 3–4 w. Fresh cells treated with SYTO-9 + propidium iodide (PI) from the Live/Dead bacterial staining kit showed green-fluorescing live bacteria along the cell periphery or dispersed in the cytoplasm (Figure 1), either stationary or moving slowly. PI, which detects dead bacteria, did not show any but stained the cell nuclei. DAPI did not stain bacteria in fresh cells but combined with SYTO-9, indicated that the latter did not stain the nucleus and that the large green fluorescing bodies resulted from blocks of SYTO-9 stained bacteria. Extended DAPI staining (60 min) detected some cytoplasmic bacteria. Following permeabilization with 0.01% TritonX-100 (30 min), SYTO-9 imparted the staining of cytoplasmic bacteria. These DNA labels normally do not stain plastids and mitochondria but in cells subjected to stronger permeabilization treatment (0.1% TritonX-100; 1 h), SYTO-9 stained these organelles, which in turn hindered the bacterial staining. 

**Figure 2:**
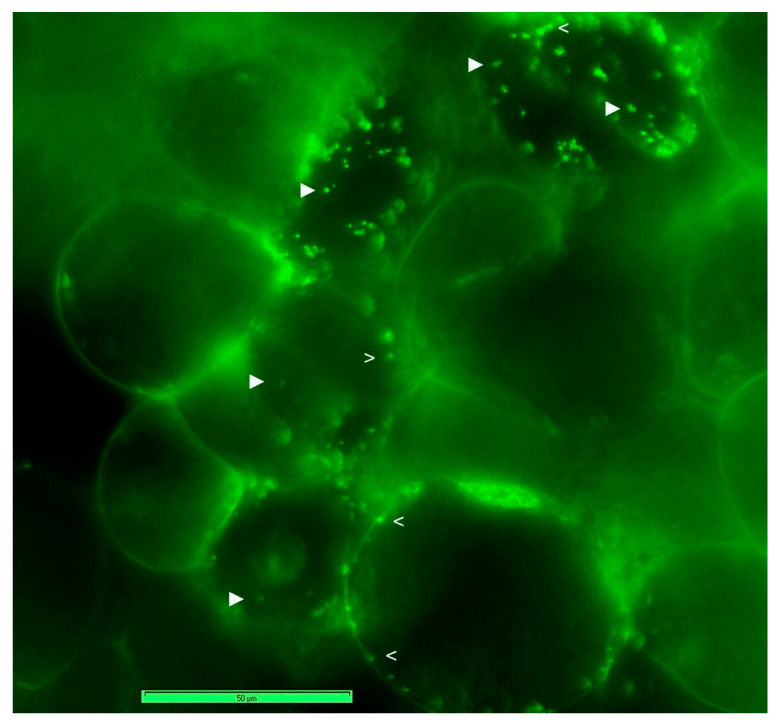
Epi-fluorescence microscopy on SYTO-9 stained grape cell culture after mild TritonX-100 permeabilization treatment (0.01%) for 30 min (40× objective). (Symbol ► indicates bacteria embedded in the cytoplasm and > shows cytoplasmic bacteria along the cell periphery.)

SYTO-9 staining of cell cultures after a mild permeabilization treatment with TritonX-100 (0.01% for 30 min) facilitated the staining of both cytoplasmic bacteria and those along the cell periphery (Figure 2), endorsing the observations on abundant motile bacteria inside the cells. The use of FDA, SYTO-9, PI and DAPI in different combinations under fluorescence microscopy indicated that the healthy 2–4 w old grape cell cultures had 95–100% cell viability; DAPI and PI stained the plant cell nucleus in a segment or all the cells depending on duration of exposure, whereas SYTO-9 stained only the bacterial cells, and the cells had ample organelles which were stained by SYTO-9, depending on cell permeabilization treatment. SYTO-9 bacterial staining did not work effectively in PBS and with formalin-fixed cells.

### 3.4. Live Imaging of Grape Cells through CLSM

**Figure 3:**
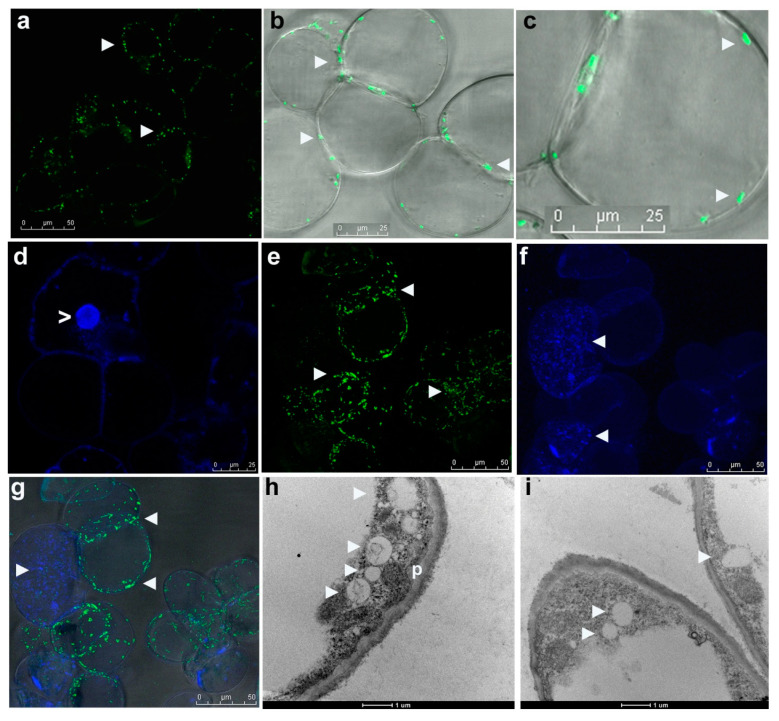
Confocal laser scanning microscopy (63× objective; (**a**–**g**)) on grape “FU01” cells stained with SYTO-9, PI or DAPI and electron microscopy (**h**,**i**). (**a**) Fresh cells stained with SYTO-9 in water show bacterial cells along the cell outline, (**b**) combined image under DIC show bacterial cells confined to the host cell along the periphery, (**c**) an enlarged view of cell with green-fluorescing bacteria, (**d**) DAPI staining of cytoplasmic bacteria and a part of perispace organisms coupled with nuclear staining, (**e**) stacking the confocal images from different cell planes over 30 µm from SYTO-9, (**f**) with DAPI or (**g**) both together displaying heavy colonization of host cells with perispace and cytoplasmic bacteria, (**h**,**i**) transmission electron microscopy showing bacterial cells in the cytoplasmic matrix as lightly stained cocci of ≤1 µm, indicated by arrowhead along with dark-stained peroxisomes of similar size marked as “p” (bar = 1 µm; symbols ► and > indicate bacterial cells and nuclei, respectively; bar length as marked in the figure).

CLSM on fresh grape suspension cells after staining with SYTO-9 + PI, SYTO-9 + DAPI or the three fluorophores singly/in combination (Leica TCS SP5 Spectral confocal system) confirmed the observations on cytoplasmic bacteria but indicated that the peripheral bacterial cells were clearly internal in the cytoplasm, lined along the plasma membrane (Figure 3a–g). The stacking of SYTO-9 images from different cell planes indicated that each host cell harbored copious bacteria endorsed by the observations with DAPI-staining of ethanol permeabilized cells. The combined SYTO-9 and DAPI image-stacks revealed bacteria in two regions; SYTO-9 mainly detected the perispace organisms, whereas DAPI stained both cytoplasmic and partly perispace bacteria. SYTO-9 usage on cellulase-treated grape cells (0.5%; 60 min) also stained the cytoplasmic bacteria. Overall, each grape cell showed heavy bacterial colonization. Direct videography on SYTO-9 stained protoplast preparation on the LSM5 LIVE confocal system showed abundant cytoplasmic and plasma membrane/nucleus-adhering bacteria (Movie S5) across different cell planes (Figure S2). No bacterial cells were observed in the host inter-cellular region.

### 3.5. Electron Microscopy

Transmission electron microscopy (TEM) on grape callus cultures showed cytoplasmic bacteria appearing as lightly stained cocci or oblong bodies (~0.5–1.0 µm) together with dark stained peroxisomes/micro-bodies (Figure 3h,i) with no apparent external membranous enclosures. Bacterial cells were seen mainly along the cell periphery, vividly cytoplasmic, and not in the periplasmic space between the cell wall and plasma membrane as originally envisioned.

### 3.6. Distinguishing Intracellular Bacteria and Plant Cell Organelles

**Figure 4:**
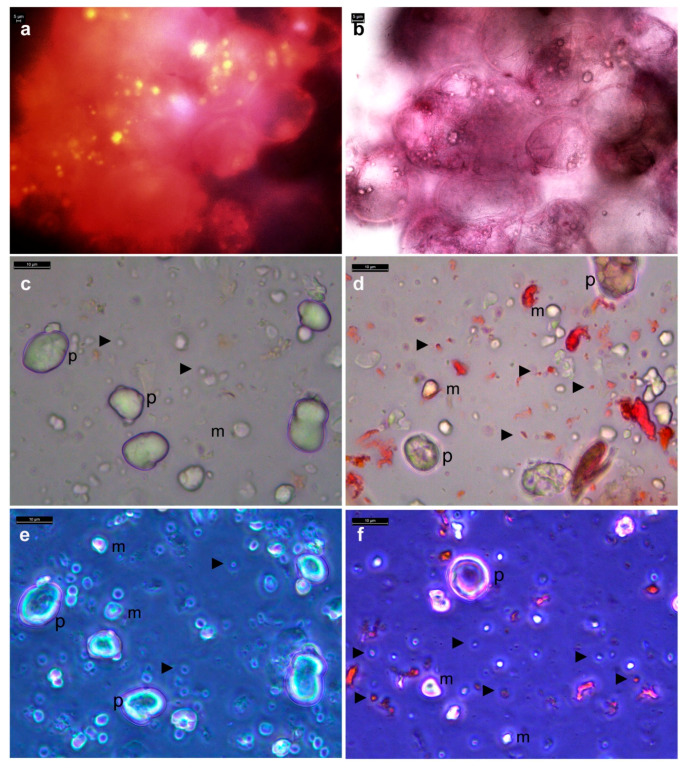
MitoTracker Orange (MTO) and DAPI staining of grape “FC01” callus cells. (**a**) Cells stained with 200 nM MTO displaying mitochondria in epi-fluorescence microscopy under N21 filter, (**b**) view of the same field under bright-field, (**c**) callus tissue homogenate at 100 mg mL^−1^ in filter-sterilized water showing plastids (p) and mitochondria (m) under bright-field or (**d**) phase contrast with abundant Cytobacts in the background (►); (**e**) tissue homogenate under bright-field or (**f**) phase contrast in the presence of 0.01% safranin showing plastids, mitochondria and Cytobacts (bar = 5 µm in (**a**,**b**) and 10 µm in (**c**–**f**) as marked).

Plastids and mitochondria, much larger than bacterial cells, were seen clearly under bright-field or with SYTO-9 staining after tissue permeabilization treatments. MitoTracker^®^ Orange (MTO) staining of fresh grape callus cells (100–200 nM) showed globoid mitochondria matching the bright-field images (Figure 4a,b). A clearer picture of plastids/mitochondria versus bacterial cells emerged from bight-field or phase-contrast microscopy on tissue homogenates directly or in the presence of safranin, showing plastids of ≥5 µm and mitochondria of ≥2 µm along with finer stained or unstained bacterial cells (Figure 4c–f). Grape cell culture treated with actin disrupting drugs that truncate peroxisome motility or the microtubule disrupting Nocodozol displayed dynamic intracellular activity, ruling out the camouflaging motility effect by micro-organelles. Further, micro-organelles devoid of DNA are not to be stained by SYTO-9/DAPI.

### 3.7. FISH on Grape Cell Cultures Using Eubacterial Probes

To confirm that the motile and SYTO-9 stained micro-particles in grape cells were indeed bacteria, FISH using eubacterial universal and class-specific probes was undertaken on “FU01” suspension culture cells. FISH required permeabilization with TritonX-100 (0.01%) for 30 min. However, FISH did not function well on formalin/paraformaldehyde/ethanol fixed cells. The use of low levels of detergents or even their avoidance during FISH was crucial for a good hybridization signal in non-fixed cells. Execution of FISH on tender cell and callus cultures was very tedious on account of the very soft and tender nature of tissues and these required very careful processing steps.

**Figure 5:**
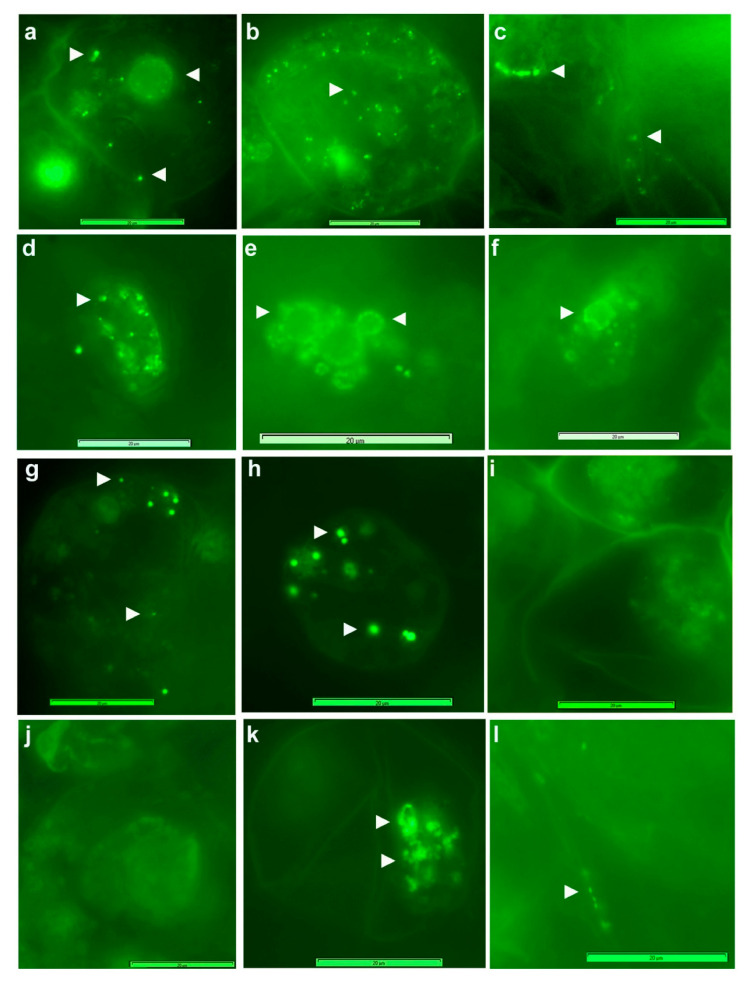
Fluorescent in situ hybridization (FISH) on grape suspension cells using FAM 5′-labeled 16S/23S rRNA gene probes (100× objective). (**a**,**b**) FISH with Eub338 probe showing intracellular bacteria in the cell matrix and around nucleus, (**c**,**d**) along the plasma membrane, and (**e**,**f**) around plant organelles; (**g**,**h**) hybridization with EubII+III detecting some larger size cocci in the cytoplasm, (**i**) cells subjected to FISH using FAM-negative control Eub338ns, (**j**) auto-fluorescence testing, (**k**,**l**) FISH with LGC354ab showing Firmicutes in cytoplasm, around the organelles and along the plasma membrane indicated by ► (bar = 20 µm). Adjustments to the images in brightness or color balance have been made to improve the bacterial detection, the extent of which is reflected in the color shade of the scale bar.

FISH with 5′-FAM-labeled probes (Figure 5) detected bacteria in the cytoplasm, along the periphery, and a significant share around the cell organelles (plastids/mitochondria/vacuoles) and nuclei with the universal Eub338 probe. The organisms appeared to be predominantly cocci or oblong to ovoid cells. No hybridization to mitochondria or plastids was observed. EubII+III, targeting other eubacteria detected some additional organisms in the cell matrix, particularly larger cocci. No signal was detected with control Eub338ns, or any significant auto-fluorescence in non-hybridized cells. LGC354ab targeting Firmicutes detected cytoplasmic bacteria and those along the cell periphery. 

**Figure 6:**
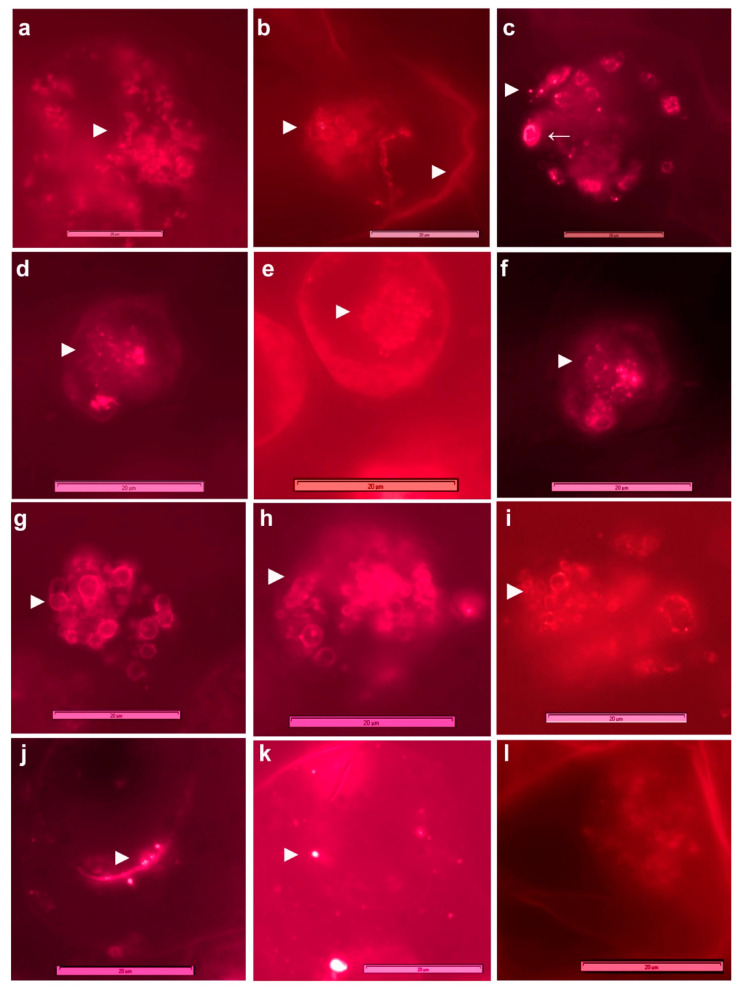
Fluorescent in situ hybridization (FISH) on grape suspension cells using Cy3 5′-labeled 16S/23S rRNA gene probes (100× objective). (**a**–**c**) Actinobacteria detected with HGC69a probe in the cell matrix, along the plasma membrane and around the cell organelles; (**d**–**f**) α-Proteobacteria with ALF969 probe in the cell matrix; (**g**–**i**) *γ*-Proteobacteria with GAM42a probe around the cell organelles, (**j**,**k**) *β*-Proteobacteria with BET42a probe seen in the cytoplasm and along the cell boundary; (**l**) cells subjected to FISH using Cy3 negative control HCG69a/ns; bacteria indicated by ► (bar = 20 µm). Adjustments to the images in brightness or color balance have been made to improve the bacterial detection extent of which is reflected in the color shade of the scale bar.

Employing Cy-3-labeled probes (Figure 6), Actinobacteria appeared abundant and were detected in the cytoplasm, along the periphery, and around organelles. Alpha and Gamma-Proteobacteria also appeared abundant, with the former seen in the cell-matrix while the latter was observed more around organelles; *β*-Proteobacteria were relatively less abundant, as confirmed subsequently with 16S rRNA gene profiling. Grape cells hybridized with control Cy3-HCG69ns did not show any fluorescence signal. 

### 3.8. Observations on Callus/Protoplast from Other Sources and Plant Species

“FC01” callus stock showed deep pink pigmentation for 1–2 months, but thereafter (3–4 months) turned brownish (Figure S3a). Yet, they showed microscopically intact cells with no cultivable bacteria detected on NA/TSA upon direct culturing or tissue homogenate plating. Freshly initiated callus cultures of grapevine (“Flame Seedless”, “Thompson Seedless”) showed white powdery or soft callus differing notably from “FC01” callus (Figure S3b). These calluses behaved identically to “FC01” during bright-field microscopy, displaying abundant motile-bacteria, but differed in cell size, obvious cellular constituents, and the configuration (size/shape) of intracellular bacteria (Movies S6 and S7). Observations on long-term maintained eight-grape callus cultures and ten medicinal plant stocks (*Catharanthus roseus*, *Ajuga reptans*, *Fallopia sachalensis*, *Polygonum cuspidatum*) also showed abundant cellular bacteria in bright-field and with SYTO-9 (Figure S3c–h). The same held well with the grape and barley callus stocks obtained from the CSIRO Plant Industry, Adelaide. The observations on freshly established callus stocks of *Arabidopsis*, tobacco (Movies S8 and S9) and protoplast preparations from the callus of the above (Movies S10 and S11) endorsed the widespread cytoplasmic bacterial association with the in vitro cultures. The absence of bacterial colony growth from the above cultures indicated the general non-cultivability of the associated organisms.

### 3.9. 16S rRNA Gene V3 Amplicon Profiling of Grape Cell Culture

**Figure 7:**
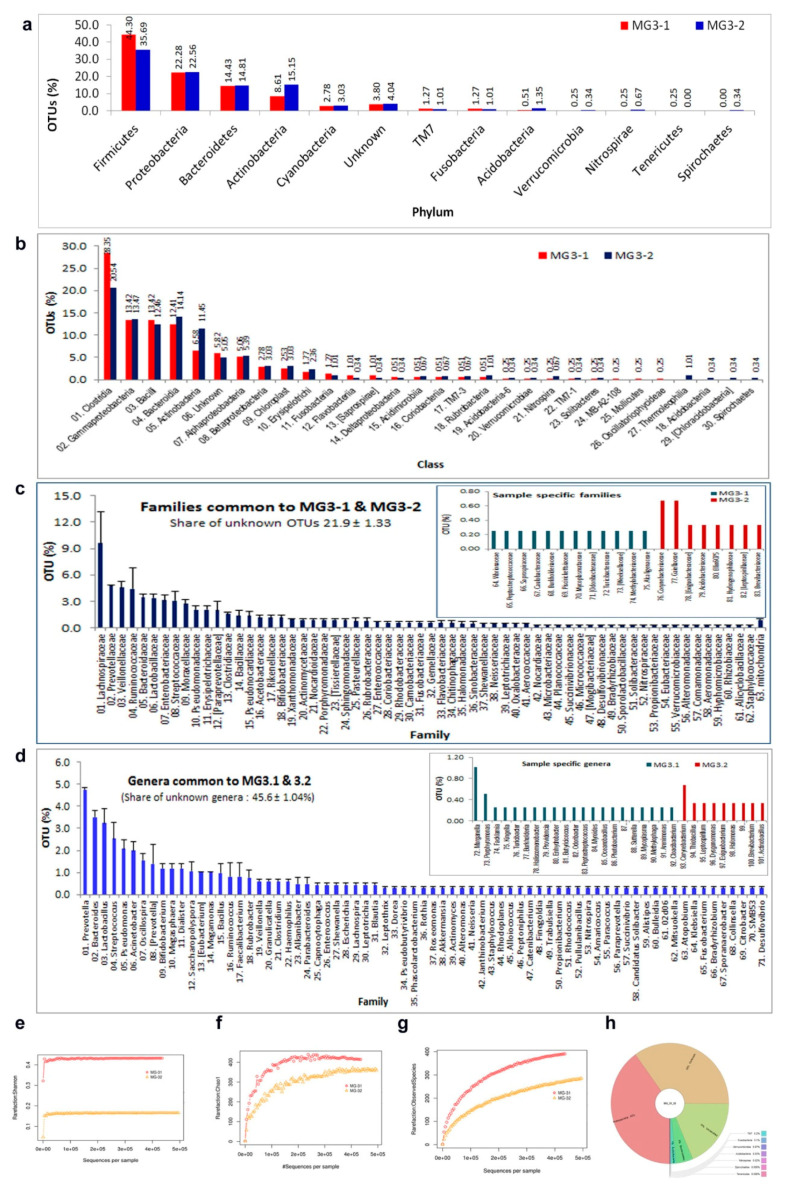
Images of 16S rRNA metagene V3 region profiling with Illumina MiSeq NGS platform on grape cell culture “FU01”-derived DNA samples MG3-1 and MG3-2 adopting 2 × 150 bp sequencing chemistry showing the distribution of OTUs after QIIME analysis-I and diversity metrics. (**a**) OTU distribution at the phylum level, (**b**) OTU distribution at the class level, (**c**), OTU distribution at the family level, and (**d**) OTU distribution at the genus level; (**e**), Shannon diversity analysis, (**f**) Chao1 diversity analysis, (**g**) observed species metrics, and (**h**) circular graphical representation of reads through Krona.

Grape “FU01” cell culture was used as a representative sample to assess the bacterial taxonomic diversity. Tissue homogenized in FDW displayed abundant bacterial cells but yielded no colony growth on NA/TSA at 30 or 37 °C. DNA isolated from two tissue homogenate samples using a MO BIO PowerFood^®^ (PF) kit (0.31 and 1.28 ng DNA µL^−1^; designated as MG3-1 and MG3-2, respectively) was used for 16S rRNA gene V3 amplicon profiling. After quality filtration steps and chimera removal, these yielded 442,020 and 554,245 PE reads with 701 and 533 OTUs, respectively (Table S2). Following singleton removal, MG3-1 and M3-2 yielded 395 and 297 OTUs, respectively. Taxonomic assignments based on QIIME showed identical OTU distribution at phylum level in both samples, with a predominance of Firmicutes (av. 40%), followed by Proteobacteria, Bacteroidetes, Actinobacteria, and Cyanobacteria (Figure 7). Both samples had minor shares of uncommon/candidate phyla (TM7, Fusobacteria, Acidobacteria, Verrucomicrobia, Nitrospirae, and Tenericutes or Spirochaetes). About 3.8–4.0% OTUs remained unassigned. Class level OTU distribution showed 26 constituents in MG3-1 and 28 in MG3-2. Clostridia formed the commonest class followed by γ-Proteobacteria, Bacteroidia, Bacilli and Actinobacteria. At order level, Clostridiales formed the major one in both samples (24.2 ± 5.59%) followed by Bacteroidales and 35–40 other orders. 

Family level OTU assignments showed 84 constituents with 76 in MG3-1, 72 in MG3-2 and 64 common to both. “Unknowns” constituted 21–23% OTUs. The major families present in both samples included Lachnospiraceae, Prevotellaceae, Veillonellaceae, and Ruminococcaceae. There were 93 and 81 assigned bacterial genera in MG3-1 and MG3-2, respectively, with 71 genera common to both the samples. About 46% of OTUs remained unassigned. Grossly, 101 genera were associated with “FU01” grape culture. Predominant genera across both samples included *Prevotella*, *Bacteroides*, *Lactobacillus*, *Streptococcus*, *Pseudomonas*, and *Acinetobacter*. 

### 3.10. 16S rRNA Gene V3–V4 Profiling on Grape Callus

PF kit derived DNA from “Gamay Freaux” “FC01” callus adopting the standard DNA extraction protocol (as for V3 amplicon profiling), or an extended lysis step of 10 min at 70 °C (2.4 and 4.1 ng DNA µL^−1^, respectively) were employed here. NGS on V3–V4 amplicons of these samples (referred to as MG11 and MG12, respectively) after stitching and chimera screening yielded 429,123 and 717,185 PE reads, respectively. QIIME-based sequence analysis after singleton removal showed 80–82% OTUs ascribed to chloroplasts and 17–19% to mitochondria. QIIME analysis-II excluding plastid, mitochondrial and unassigned sequences, and quality filtration steps (Table S3) yielded 455 and 581 OTUs, respectively. 

**Figure 8:**
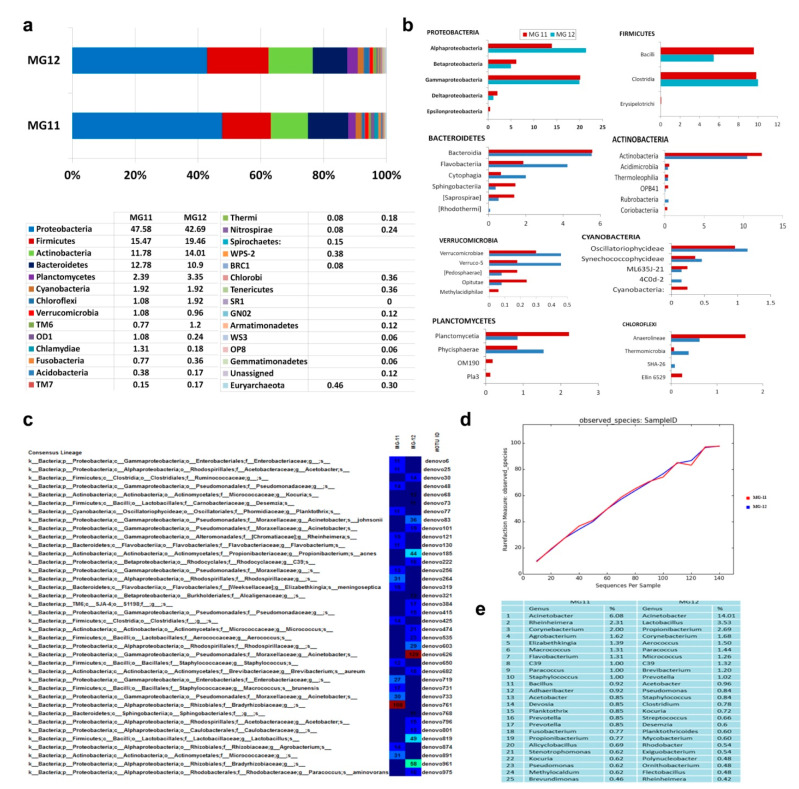
Images of 16S rRNA metagene V3–V4 region profiling with Illumina MiSeq NGS platform with 2 × 300 bp sequencing chemistry on grape callus “FC01”-derived DNA samples MG11 and MG12. (**a**) Distribution of OTUs under different phyla in MG11 and MG12 samples after QIIME analysis-II (excluding the chloroplast, mitochondrial and unassigned sequences) displaying high extent of bacterial diversity, (**b**) Predominant Classes under different phyla in MG11 and MG12 samples, (**c**) OTU Consensus lineage for MG11 and MG12 samples, (**d**) Rarefaction plot, (**e**) Top 25 genera in MG11 and MG12 samples.

Phylum level taxonomic distribution showed 21 phyla in MG11 and 26 in MG12, which included several candidate phyla and one phylum each under Euryarchaeota (Figure 8a). Proteobacteria formed the predominant phylum in both, followed by Firmicutes, Actinobacteria, Bacteroidetes, Planctomycetes, and Cyanobacteria. The rest, in lower amounts, were distributed under 15 phyla in MG11 and 19 phyla in MG12, besides one under Archaea in both. At the class level, MG11 showed a predominance of α- and γ-Proteobacteria with relatively low proportions of β-, δ- and ε-Proteobacteria (Figure 8b). Firmicutes included Clostridia and Bacilli. Bacteroidetes were comprised of Bacteroidia, Flavobacteria, Cytophagia, Saprospirae and Sphingobacteria. Phylum Actinobacteria constituted the classes Actinobacteria, Acidimicrobiia, Thermoleophilia, OPB41 and Rubrobacteria. Planctomycetes included Phycisphaerae and Planctomycetia. The rest of OTUs represented 25 classes under 15 phyla. It was striking to note two classes under Euryarchaeota (Methanobacteria and Methanomicrobia) and several candidate phyla with no cultured relatives in small shares (OD1, TM6, TM7, WPS-2, SC4, BRC1, Thermi, Acidobacteria, Fusobacteria). The extended extraction procedure in the MG12 sample revealed an additional 22 classes over MG11, including eight candidate phyla. Thus, the 16S rRNA gene taxonomic profiling revealed an enormous diversity of cultivation-recalcitrant bacteria in grape callus stock. 

The consensus lineage as per OTU heat-map in MG11 showed Bradyrhizobiaceae under α-Proteobacteria and Micrococcaceae under Actinobacteria as the predominant families, while MG12 showed Moraxellaceae as the predominant group followed by Bradyrhizobiaceae. At the genus level, the OTUs in MG11 were distributed across 223 genera, of which 126 could be clearly described with cultured representatives (Dataset S1) while MG12 showed 264 genera with 131 assigned to specific genus (Dataset S2). The predominant assigned genera showed most of them common to both samples. Alpha diversity analysis suggested the presence of 455 species in MG11 and 581 in MG12, with rarefaction analysis indicating the recovery of a large share of species diversity (Figure 8c–e). The predominant genera derived through V3–V4 profiling showed considerable similarity between the two replicate samples as for the V3 profiling. However, the genera deciphered through the two approaches differed to some extent (Dataset S3), which may also be linked to the cell culture/callus stocks.

A comparison of taxonomic diversity between 40+-year-old “Gamay Freaux” callus and the newer callus stocks of “Flame Seedless” (MG18) and “Thompson Seedless” (MG19) generated in another study showed some common and some distinct genera (Figure S4). “Gamay Fréaux” showed more diversity (131 genera) compared with “Flame Seedless” (90 genera) and “Thompson Seedless” (104 genera). About 44 genera were common to all three samples, with 70, 11 and 20 unique genera, respectively, in the above cultivars. 

### 3.11. Activation of Uncultured Bacteria from “FU01” Cells to Cultivation

Based on the observation from grape tissue taxonomic profiling that most of the associated organisms belonged to genera that are often cultivated, their culturing feasibility was attempted. HTH from grape FU01 (100 mg mL^−1^ FDW) incubated in 0.1× nutrient broth or 0.1x tryptone soya broth for a week (27/30 °C) yielded four organisms upon plating on NA/TSA. These were identified based on 16S rRNA gene homology analysis as *Bradyrhizobium elkanii*, *Staphylococcus pasteuri*, *Sphingomonas paucimobilis* and *Bacillus megaterium* (NCBI Acc nos. KC787046–KC787049). The first three organisms with small cocci or rods appeared quite similar in size and shape to the motile microparticles observed in the host cells and HTH. The share of activated bacteria constituted about 2.9% of the total genera deciphered through 16S rRNA V3 amplicon profiling or about 1.1% from V3–V4 profiling. These organisms, upon reintroduction to grape cultures, grew as obvious contaminants soon or after one passage, suggesting that the cultivable stage in culture medium was deleterious to the in vitro stocks. 

### 3.12. PICRUSt Functional Analysis on 16S rRNA Gene V3–V4 Data

**Figure 9:**
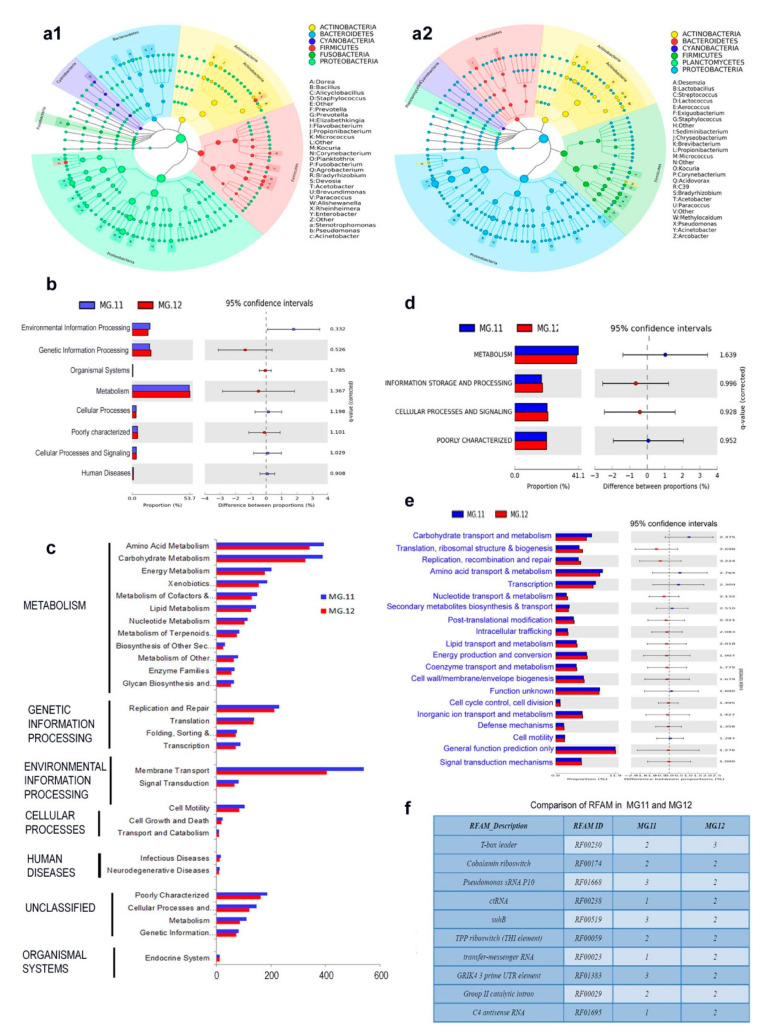
PICRUSt mediated endophytic bacterial functional analysis on 16S rRNA metagene V3–V4 profiled FC01 grape callus. (**a1**,**a2**) Cladogram showing the taxonomic diversity from phylum to genus level based on defined genera in “Gamay Fréaux” MG11 and MG12 samples, (**b**) functional analysis based on KEGG pathway Level 1 and (**c**) Level 2, (**d**) COG-based functional analysis at Level 1 and (**e**) Level 2 and (**f**) R-FAM analysis in MG11 and MG12.

Taxonomic classification based on defined genera confirmed Proteobacteria as the predominant phylum followed by Actinobacteria and Firmicutes (Figure 9a). PICRUSt-based functional analysis showed metabolism as the most abundant functional gene category (Level 1) as per the KEGG pathways, followed by genetic information processing. The most abundantly represented functional gene families (Level 2) included membrane transport, followed by amino acid metabolism, carbohydrate metabolism, and others (Figure 9b,c). Level 3 functional roles included 227 categories across MG11 and MG12 samples. The major KEGG Orthology groups (KOs) included iron complex outer membrane receptor protein, enoyl-CoA hydratase, RNA polymerase sigma-70 factor, ECF subfamily, etc., with the total KOs spanning across >1500 groups (data not shown). Abundances of COGs also showed metabolism as the major functional gene category (Level 1), followed by cellular processes and signaling, and information storage and processing (Figure 9d,e). The most abundantly represented functional gene families (Level 2) included general function prediction followed by amino acid transport and metabolism, transcription, energy production and others. It was also worth noting several noncoding RNA families as per the RFAM analysis (Figure 9f).

### 3.13. Microscopic Observations on Field Plant Tissues

Grape shoot and petioles sections showed motile bacteria in the cell lumen (Movie S12) in both cultivars. Cell scanning on the x–y and x–z planes showed them in most cells with some cell-to-cell variation in their extent and size. Microscopy on seedling hypocotyl and shoot/petiole tissues of other callus-source plants—*Arabidopsis*, tobacco and periwinkle—confirmed the prevalence of abundant intracellular bacteria (Movies S13–S15), indicating their carrying over through surface-sterilized tissues and their inevitable in vitro introduction.

## 4. Discussion

Healthy plant cells are normally not considered to bear another life form. Conversely, this study unearths the prevalence of abundant cultivation-recalcitrant endophytic bacteria (CREB) as intracellular inhabitants in live plant cells grown in vitro for varying periods, brought out principally through live-cell microscopy on healthy cell/callus cultures and verified further through eubacterial FISH and molecular diversity analyses. The long-term cultured plant cell and callus cultures displayed such diverse CREBs in abundance with their origin ascribable to the field tissues. The tissue culture system, with elaborate sterility checks, provided a clear sterile arena protected from external microorganisms. Microscopy, coupled with the cultivation-based and cultivation-independent bacterial monitoring tools, indicated that the organisms were not easily cultivable. It was likely that any associated cultivable endophytic microorganisms were excluded at culture initiation with their manifestation as contaminated stocks. The consistency with several cell and callus lines belonging to various plant species initiated independently in different laboratories from diverse plant organs, and maintained for varying periods as healthy cultures indicated that the intracellular bacterial association is a widespread phenomenon in cultured plant cells. The observations on intracellular bacteria with the field/seedling tissues of callus-source plants in this study indicated the prevalence of such associations in field plants and their possible in vitro introduction through surface-sterilized shoot tissues. This finding is endorsed by intracellular bacteria documented with Scotch pine meristem [23], shoot-tip tissues, and in vitro cultures of bananas [25] and papaya [26], micropropagated pineapple and orchids [24], and the nuclear-adhering *Methylobacterium extorquens* in pine [46]. The term “Cytobacts” is proposed to describe such cytoplasmic bacteria in line with the intracellular bacteria documented in banana [25] to facilitate their further description and characterization. 

The general conception surrounding bacterial endophytes is that they get recruited by plants, mainly from soil or occasionally from aerial plant parts [2,3,12]. From soil, the plant entry is mostly through root hairs/rootlets [47,48,49]. They further sail through the root cortex, entering the vascular system with subsequent inter-cellular or apoplastic colonization in different plant parts/organs [2,3,49]. It is intriguing how the organisms gain cytoplasmic entry, bypassing the host defense mechanisms. One way is that this could arise from the in vitro cell proliferation from vascular tissues. On the other hand, the reports cited above [23,24,25] and our recent observations on intracellular bacterial association as a widespread phenomenon across plant species, based on a survey of taxonomically and morphologically diverse field plants, suggests “Cytobacts” is a ubiquitous phenomenon among vascular plants (Thomas et al., unpublished results), [50]. It is also contentious how this ubiquitous association prevailing in tissue cultures went undetected for so long. This could be due to the general uncultivability of the associated organisms, the broad assumption of microbial freedom of healthy plant cells, the aseptic and axenic characteristic of tissue cultures, and/or the lack of concerted efforts by researchers. Such intracellular micro-particle motility was perhaps overlooked as “Brownian motion” [51], micro-organelle movement [37,38,52], or mere cytoplasmic streaming [53]. The hallmark of this study has been the application of simple live-cell video-microscopy on intact cell and protoplast cultures and the use of fresh tissues for fluorescence/confocal microscopy on which SYTO-9 bacterial staining and 16S rRNA-mediated FISH-technique worked well. On the other hand, the conventionally employed fixed tissues failed to bring out the bacterial associations. SYTO-9 provided the crucial first line evidence for abundant cytoplasmic organisms further endorsed with TEM, FISH, and 16S rRNA metagene taxonomic and functional profiling. 

An earlier report on endophytic bacteria in banana suggested two niches of cellular colonization, namely cytoplasmic and periplasmic, the latter in between the cell wall and plasma membrane as per the epi-fluorescence microscopic observations [36]. CLSM observations on fresh grape suspension cells in this study, supported by TEM, confirmed the observations on cytoplasmic colonization [25], and also indicated that the peripheral bacterial cells were also cytoplasmic, adhering internally to the plasma membrane. It was also common to observe the endophytic bacteria adhering to cell organelles, such as mitochondria, plastids and vacuoles, and the nucleus. Some bacterial motility was also observed inside the vacuoles. 

Another significant aspect of this study has been the elucidation of unprecedented taxonomic diversity harbored by 40+ year-old grape cell/callus cultures. Such an extent of bacterial diversity has been documented for roots in NGS-based studies [8,9,54]. Recent NGS-mediated explorations on olive, banana, and papaya have also indicated immense bacterial diversity prevailing in shoot tissues [11,26,31]. This has been endorsed with the field shoot tissues and freshly established callus tissues of grapevine, which also formed the subjects of present investigation [22]. One major limitation while applying cultivation-independent approaches to study endophytic bacteria was the interference from chloroplast and mitochondrial sequences, which are of Prokaryotic lineage [8]. QIIME offered the solution of filtering out such intrusive reads, amounting to >95% gross reads, thereby bringing out the high taxonomic variability. Proteobacteria constituted the major phylum as per 16S rRNA V3–V4 profiling, as documented with the roots of *Arabidopsis* [8] and rice [9], and the shoot tissues of banana [11] and grapevine [22] with a predominance of α-and γ-classes, followed by Firmicutes and Actinobacteria, consistent with the FISH data. These three phyla form the commonly documented endophytes in field plants. It was significant to record substantial shares of Bacteroidetes and Planctomycetes in grape cell cultures besides a series of uncommon and candidate phyla that lack cultured representatives [55]. 

An earlier 16S rRNA taxonomic profiling study comparing the endophytic bacterial microbiome of field shoot-tip tissues of grapevine cv. “Flame Seedless” and “Thompson Seedless” versus freshly initiated callus stocks from shoot-tips displayed broad bacterial diversity in callus cultures bearing a resemblance to field tissues with Proteobacterial dominance [22]. In addition to endorsing the field origin of the endophytic bacterial microbiome, this also indicated some taxonomic realignment during in vitro tissue growth. Thus, callus tissues displayed a reduction in the share of Proteobacteria, the enrichment of Actinobacteria and Firmicutes, the disappearance of some field-associated phyla, and the emergence of a few additional taxonomic groups over field community. Further, a comparison of phylogenetic taxa in long-term maintained grape “FC01” callus (MG12) with the more recently initiated callus stocks of grapevine (MG18 and MG19, respectively, as per [22], showed notable differences in the profiles possibly linked to the long-term in vitro culture maintenance or genotypic differences.

It was also significant to document some Archaea under the phylum Euryarchaeota in long-term maintained grapevine “FC01” callus cultures under the classes Methanomicrobia and Methanobacteria, albeit in low shares. Archaea as root endophytes in maize and rice were documented based on initial cultivation-independent studies [6,56]. The 16S rRNA gene amplicon sequencing approach on leaf tissues of *Olea europaea* L. changed this perception, exhibiting a high portion of archaeal taxa under the phyla Thaumarchaeota, Crenarchaeota, and Euryarchaeota [57]. Of note, 16S rRNA amplicon studies with more recently initiated grape callus tissues showed a minor share of archaea [22], but a more significant share (5%) was documented in tomato seeds [58]. Archaeome is now increasingly recognized as an essential component of host-associated microbiomes, including plants [59].

The absence of bacterial colony growth from the cells/tissues or the culture medium of long-term well-maintained callus and cell cultures on standard bacteriological media and additional test media versus the microscopic detection of profuse bacterial cells or the high 16S rRNA gene taxonomic diversity suggested that the associated organisms were not easily cultivable. On the other hand, it was possible to activate a few organisms, which are known to be endophytes of different plants (*Bradyrhizobium elkanii*, *Staphylococcus pasteuri*, *Sphingomonas paucimobilis* and *Bacillus megaterium*) from grape cell cultures. Additionally, 16S profiling (MG11 and MG12) also showed *Bradyrhizobium* as a dominant genus, also documenting the other three genera. The bacterial activation to cultivation was facilitated with the host-tissue-homogenate incubation in a low-nutrient medium as done earlier with banana and watermelon tissues [44,60]. This indicated that, with the provision of proper growing conditions, it was feasible to trigger some of the cultivation-shy organisms to cultivate. Therefore, the term cultivation-recalcitrant endophytic bacteria (CREB) appeared more suitable to describe such endophytic bacteria [14,58]. 

The “in cyto” survival of various organisms in long-term actively maintained cell and callus cultures without any apparent adverse effects suggested that the organisms share a mutualistic relationship with the hosts. Endophytic bacteria have been known to have several beneficial properties in native plants and with lateral introduction, including plant growth promotion, biotic and abiotic stress alleviation, and production of secondary metabolites [2,15,61]. PICRUSt-based functional analysis on grape “FC01” callus stock indicated diverse roles for the endophytic bacterial community with metabolic pathways as the major part. Thus, several pathways that are considered as plant-derived could actually be of microbial origin or mediated by the endophytic microorganisms [25,62]. The unique/distinct features shown by the respective callus tissues could be linked to the associated organisms. The pink pigmented “Gamay Fréaux” culture is highly valued for anthocyanin biosynthesis [32], and it showed some distinct differences in the taxonomic profile compared to the non-pigmented calluses of “Flame Seedless” and “Thompson Seedless”. This implies that some of the associated organisms or a bacterial consortium could be involved in the metabolic pathways. The observations have significant implications, considering the widespread usage of plant cell cultures for deciphering metabolic pathways and the production of novel biomolecules, especially anthocyanin biosynthesis studies in this instance [32,34]. In the absence of recognizing the microbial association within cell cultures, their activities, pathways, and products get viewed as normal properties of host plant cells, as was proposed for *Methylobacterium* associated with the callus cultures [62]. The association of an array of organisms and their general non-cultivability made the elucidation of the functional roles of various phylogenetic groups or their constituents a tedious task. So far, only very limited studies have been documented on functional elucidation of intracellular bacteria which again is confined to one cultivable bacterium of *Methylobacterium* sp. in pine [23,46]. It would warrant specific antibiotic challenge assays to eliminate or cull different organisms to get clearer functional information on various organisms calling for bigger team efforts. 

It is possible that the cultivation-recalcitrance of these bacteria is compounded by their protracted obligate association in cell cultures or their genome modification/reduction, as documented with insect endosymbionts [63] or members of uncultured candidate phyla [55]. It calls for detailed investigations to understand the nature of associations between endophytes and the plant hosts, following the threads with endosymbionts and their animal hosts [64,65]. 

The current observations on intracellular bacteria suggest that they could move to the daughter cells at cell division through mitosis and meiosis, during which the cytoplasmic constituents get distributed to the daughter cells. This strengthens the possibility of vertical transmission of endophytes through the gametes and seeds. The seed transmission of endophytic bacteria is a topic of great interest now [16,66], but it is often questioned if the organisms are true embryo colonizers [58]. A recent study using watermelon seed-embryos, which could be excised distinctly, excluding the seed-coat tissues, indicated the vertical transmission of diverse cultivation-recalcitrant endophytic bacteria, which is consistent with the intracellular colonization (Thomas, unpublished results [67]). The cytoplasmic bacterial association facilitates their vertical transmission across generations, also explaining the possibility of their continuous presence as intracellular inhabitants. The current observations also assume evolutionary significance in the context of serial endosymbiotic theory, which suggests that plastids and mitochondria originated, perhaps by symbioses, with the third type of microbe [68]. A vivid understanding of the cell constituents and their functioning is a requisite in plant cell biology. The unearthing of the ubiquitous existence of an “alien life” in long-term actively maintained live plant cells with their possible origin in field tissues and possible vertical transmission opens the gateway for further research on various interrelated aspects.

## 5. Conclusions

This study unearths the prevalence of abundant and diverse cultivation-recalcitrant endophytic bacteria (CREB) in healthy plant cells, employing the freshly initiated as well as long-term actively maintained cell and callus cultures across diverse plant species anchoring on microscopic and molecular evidence. The organisms appeared to be present in a cultivation-recalcitrant form, largely colonizing the cytoplasm and thus described as “Cytobacts”. Of note, 16S rRNA gene sequence-based molecular diversity analysis on long-term actively maintained grapevine cell and callus-cultures indicated a huge taxonomic diversity comprising nearly 99% eubacteria spanning >15 phyla and a minor share of Archaea. A preliminary search for the potential source of these intracellular bacteria in tissue cultures pointed to the prevalence of such intracellular bacteria in field plants and the in vitro entry through surface-sterilized shoot-tissues.

## Data Availability

16S rRNA sequence data of bacteria isolates have been deposited with the NCBI Genbank (acc. nos. KC787046- KC787049) and the 16S rRNA metabiome with NCBI-SRA (Bioproject ID: PRJNA286272). P.T. may be contacted regarding data and C.M.M.F. for cell cultures maintained at Flinders University.

## Supplementary Materials

The following are available online at https://www.mdpi.com/2076-2607/9/2/269/s1. Table S1. Description of cell suspension and callus cultures used for the microbial association investigations along with the indexing media and conditions employed for detecting cultivable bacteria; Table S2. Data on 16S rRNA metagene V3 profiling for MG3-1 and MG3-2 samples derived through Illumina MiSeq and phylogenetic distribution of OTUs, as per direct QIIME analysis (QIIME round-1) of 16S rRNA V3 sequence data; Table S3. Data on 16S rRNA metagene V3–V4 profiling for MG11 and MG12 samples derived through Illumina MiSeq and QIIME analysis of sequence data directly, and after excluding chloroplast, mitochondrial and unassigned sequences showing the distribution of OTUs under different phyla; Figure S1. Bright-field microscopy on non-fixed “FU01” grape suspension culture. (a) Cells under 100× magnification appearing in small clusters (20–40 µm) with red-pigmented patches under low magnification; (b) at 400× magnification displaying plastids and mitochondria as larger irregular or circular objects; (c–f) at 1000× magnification; (c) cells fixed in 4% formalin showing abundant grainy non-motile micro-particles that correspond to cellular bacteria; (d,e) formalin-fixed cells stained with 0.005% safranin displaying pink stained bacterial cells with larger plastids and mitochondria; (f) an intact grape cell stained with safranin displaying bacterial cells adhering to organelles (bar = 10 µm); Figure S2. LSM5 live Confocal z-scan images (at 1 µm) on grape cell culture “FU01”. Isolated grape cell derived after cellulase treatment stained with SYTO-9 displaying green fluorescing bacteria in the cytoplasm and along the perispace and adhering to the nucleus (bar = 5 µm); Figure S3. Callus stocks from different plant sources and laboratories in direct view or with epi-fluorescence microscopy after SYTO-9 staining. (a) Grape “FC01” callus on MS-agar-based callusing medium after one, two or four months of culturing (left to right), (b) one-month-old callus cultures of “Gamay Freaux” “FC01”, “Flame Seedless”, and “Thompson Seedless” (left to right), (c) callus stock “FC-04” of grape “Pinot Noir”, (d) “Chardonnay”, and (e) “Cabernet Sauvignon”, (f) callus stock of *Catharanthus roseus*, (g) callus stock of barley “Golden Promise” and (h) callus stock of barley “Sloop” (Bar = 50 µm); Figure S4. VENN diagram showing the distribution of phylogenetic taxa in callus tissues of three grape cultivars “Gamay Freaux” (GF), “Flame Seedless” (FS) and “Thompson Seedless” (TS) at different taxonomic levels. (a) Common/distinct taxa defined at phylum, (b) class, (c) order, (d) family and (e) genus levels based on gross diversity; (f) list of common genera in the three callus stocks; (g) unique genera in “Gamay Freaux’; (h) “Flame Seedless”; (i) “Thompson Seedless”; (f–i) are based on defined genera only. (A comparison of defined genera present in the three callus tissue samples showed 44 common to all three samples; 6 genera common to “Gamay Freaux” and “Flame Seedless”, 10 genera common to “Gamay Freaux” and “Thompson Seedless”; 30 genera common to “Flame Seedless” and “Thompson Seedless”. In total, 70 genera appeared unique to “Gamay Freaux”, 11 unique to “Flame Seedless”, and 20 genera unique to “Thompson Seedless”); Dataset S1. Genus level abundance of grape callus bacterial OTUs derived through 16S rRNA gene amplicon profiling of sample MG11; Dataset S2. Genus level abundance of grape callus “Gamay Freaux” bacterial OTUs derived through 16S rRNA gene amplicon profiling of sample MG12; Dataset S3. Genus level OTU distribution (%) based on 16S rRNA gene V3 or V3–V4 region amplicon profiling of gape cell/callus culture; Movie S1. Fresh grape suspension “FU01” cells examined under bright-field in 0.005% safranin showing actively motile intracellular bacteria in substantial numbers in different x–z planes (1000×); Movie S2. Grape suspension “FU01”-separated fresh cells under bright-field showing actively motile intracellular bacteria in the cytoplasmic matrix in different x–y fields (1000×); Movie S3. Grape suspension “FC01” single intact cell showing abundant intracellular and intra-vacuolar bacteria (1000× bright field); Movie S4. Pure culture of *Staphylococcus pasteuri* activated from grape cell suspension culture showing the active movement of cocci in a liquid film similar to grape intracellular bacteria (1000× bright field); Movie S5. LSM5 LIVE Confocal z-scan images on grape “FC01”-isolated cells, derived after cellulase treatment displaying green fluorescing bacteria in the cytoplasm, along the cell periphery and adhering to the nucleus after staining with SYTO-9; Movie S6. Grape “Flame Seedless” callus cells under bright-field, showing actively motile intracellular bacteria in the cytoplasmic matrix in different vertical fields (1000×); Movie S7. Grape “Thompson Seedless” callus cells under bright-field, showing actively motile intracellular bacteria in the cytoplasmic matrix in different vertical fields (1000×); Movie S8. Callus stock of *Arabidopsis* under bright-field showing actively motile intracellular bacteria in the cytoplasmic matrix in different vertical x–z fields (1000×); Movie S9. Callus stock of tobacco under bright-field showing actively motile intracellular bacteria in the cytoplasmic matrix in different vertical fields (1000×); Movie S10. Enzymatically isolated cell from callus stock of *Arabidopsis* under bright-field showing actively motile intracellular bacteria in different vertical fields (1000×); Movie S11. Protoplast preparation from callus stock of tobacco under bright-field showing actively motile intracellular and intra-vacuolar bacteria in different vertical fields (1000×); Movie S12. Bright-field microscopy on fresh tissue section from surface-sterilized tender shoot-tip tissue of field-grown grape “Flame Seedless” showing intracellular bacteria (1000×); Movie S13. Bright-field microscopy on intact hypocotyl tissue of surface-sterilized 1 w old *Arabidopsis thaliana* soil-grown seedling showing intracellular bacteria (1000×); Movie S14. Bright-field microscopy on fresh tissue section from surface-sterilized petiole tissue of glasshouse-grown tobacco plant showing intracellular bacteria (1000×); Movie S15. Bright-field microscopy on fresh tissue section from surface-sterilized petiole tissue of field-grown *Catharanthus roseus* plant showing intracellular bacteria (1000×).

## Abbreviations

CLSM	confocal laser scanning microscopy
FDW	filter sterilized distilled water post-autoclaving
NA	nutrient agar
TEM	transmission electron microscopy
HTH	host tissue homogenate
TSA	tryptone soy agar
