# Supplementary material for: Intracellular Bacteria in Plants: Elucidation of Abundant and Diverse Cytoplasmic Bacteria in Healthy Plant Cells Using In Vitro Cell and Callus Cultures
Source: Microorganisms. 2021 Jan 28;9(2):269. doi: 10.3390/microorganisms9020269 (PMC7912260; doi:10.3390/microorganisms9020269)
Supplement: Supplementary file 1 [file microorganisms-09-00269-s001.zip › Supplementary material of microorganisms-1079905-Final version/Supplementary Tables and Figures.pdf]

Table S1. Description of cell suspension and callus cultures used for the microbial association investigations along with the indexing media and conditions employed for detecting cultivable bacteria.

Cell line ID	Species (Variety/ common name)	Source of material	Remarks on cultures	Indexing media /conditions employed
A. Cell suspension / callus cultures maintained at Flinders university				
i. Cell suspension cultures				
FU-01	Grapevine *Vitis vinifera* L. (Gamay Freaux)	Suspension initiated at Flinders (2000) from callus originated in 1978 (Cormier et al. 1990)	Initially pink pigmented, turning purple	NA (pH 5.5/7.4; 27/37°C), TSA (pH 5.5/7.4; 27/37°C), BHIA (pH 7.4; 30°C), TF1, TF2, TF3, TF4 (pH 6.0; 30°C)
FU-04	*Vitis vinifera* L. (Gamay Freaux)	-do-	Fast growing, high anthocyanin selection	-do-
ii.. Callus cultures				
FC-01	*Vitis vinifera* L. (Gamay Freaux)	Callus originated in 1978 at Quebec, Canada (Cormier et al. 1990) Maintained at Flinders from 2000.	Range of pigment levels	NA (pH 5.5/7.4; 37°C), TSA (pH 5.5/7.4; 30°C), TF4 (pH 6.0; 30/37°C)
FC-02	*Vitis vinifera* L. (Gamay Freaux)	Low pigment microcallus selected from FC-01 (Flinders, 2002)	No visible pigment	-do-
FC-03	*Vitis vinifera* L. (Gamay Freaux)	High pigment microcallus from FC-01 (Flinders, 2002)	High anthocyanin	-do-
FC-04	*Vitis vinifera* L. (Pinot Noir)	Initiated at Flinders before 2001	Vigorous growth, low pigment	-do-
FC-05	*Vitis vinifera* L.	CSIRO Food Science,	Medium	-do-

	(Pinot Noir)	Sydney (recd. 2002)	pigmentation and consistent levels	
FC-06	*Vitis vinifera* L. (Pinot Noir)	CSIRO Food Science, Sydney (recd. 2002)	Pigment levels vary over time	-do-
FC-07	*Vitis vinifera* L. (Chardonnay)	Leaf (Flinders, 2003)	Low pigment; Vigorous growth	-do-
FC-08	*Vitis vinifera* L. (Shiraz)	Berry (Flinders, 2003)	Light pigmented/ variable	-do-
FC-10	*Ajuga reptans* L.	AlfonsCallebaut, Belgium (recd. 2004)	Deep purple, fast growing	NA (pH 7.4; 37°C), TSA (pH 7.4; 30°C), TF4 (pH 7.4; 30°C)
FC-11	*Fallopia sachalensis* F. Schmidt (Japanese knotweed)	Leaf (CSIRO Food Science, Sydney, recd. 2004)	Brownish, soft type	-do-
FC-12	*Fallopia japonica* (Houtt.) Ronse Decr. (Japanese knotweed)	Stem / leaf (CSIRO Food Science, Sydney (recd. 2004)	Soft and a little crumbly callus	-do-
FC-13	*Fallopia japonica*	Leaf (CSIRO Food Science, Sydney (recd. 2004)	Very slow growing; cream with brown spots	-do-
FC-14	*Polygonum cuspidatum* Siebold&Zucc. (Knotweed)	Stem (Flinders, 2004)	Cream yellow vigorous growing	-do-
FC-17	*Catharanthus roseus* (L.) G.Don (Periwinkle)	Stem (Flinders, 2005)	Caramel cream	-do-
FC-19	*Catharanthus roseus*	Leaf (Flinders, 2005)	Pale cream	-do-
FC-20	*Catharanthus roseus*	Leaf (Flinders, 2005)	Brown	-do-
FC-21	*Catharanthus roseus*	Nodes (Flinders, 2005)	Yellowish brown	-do-

FC-22	*Catharanthus roseus*	Leaf (Flinders, 2005)	Dark tan/ black	-do-
B. In vitro stocks obtained from other sources for this study				
Callus cultures from CSIRO Plant Industry, Adelaide				
1.	*Vitis vinfera* L. (Cabernet sauvignon)	*In vitro* leaf (Original source: Adelaide University, 2001)	White, soft, fast growing	NA (pH 7.4; 37°C), TSA (pH 7.4; 30°C), BHIA (pH 7.4; 30°C)
2.	*Vitis vinifera* L. (Chardonnay)	*In vitro* stem/petiole, (CSIRO Plant Industry, 2002)	White, soft, fast growing	-do-
3.	*Vitis vinifera* L. (Pinot Noir)	(Original source: Flinders, 2002)	White, soft, fast growing	-do-
4.	*Vitis vinifera* L. (Shiraz)	*In vitro* stem/petiole, (CSIRO Plant Industry, 2002)	White, soft, fast growing	-do-
5.	*Vitis vinifera* L. (Shiraz)	Berry (CSIRO Plant Industry, 2002)	Visibly firm but friable; fast growing	-do-
Callus cultures from School of Botany, The University of Melbourne				
1.	*Hordeum vulgare* L. (Golden Promise)	In culture for 4 years	Soft callus	NA (pH 7.4; 37°C), TSA (pH 7.4; 30°C)
2.	*Hordeum vulgare* L. (Sloop)	In culture for >5 years	Soft callus	-do-
3.	*Hordeum vulgare* L. (Schooner)	In culture for >5 years	Soft callus	-do-
Newly initiated cultures at the Indian Institute of Horticultural Research, Bangalore				
1.	*Vitis vinifera* L. (Flame Seedless)	Internode and petiole; 6-12 months	Medium form light pink callus	NA (pH 7; 37°C), TSA (pH 7; 30°C)
2.	*Vitis vinifera* L. (Thompson Seedless)	Internode and petiole; 6-12 months	Soft white callus	-do-
	Musa sp. Banana (Grand Naine)			
3	*Nicotiana tabacum;*	Leaf and petiole; 2-	Fine to coarse	-do-

	cell suspension culture	4 months	suspension	
4.	*Nicotiana tabacum* (L.) callus	Leaf and petiole; 3-6 months	Soft callus	
5.	*Arabidopsis thaliana* (L.) Heynh. callus	Internode and petiole; 6 months	Firm callus	-do-

Abbreviations : NA, nutrient agar; TSA, trypticase soy agar, BHIA, brain-heart infusion agar, TF 1, 2, 3, 4: Combinations involving one-tenth each of NA & TSA (TF 1), NA & BHIA (TF 2), TSA & BHIA (TF 3) or NA, TSA & BHIA (TF 4).

Table S2. Data on 16S rRNA metagene V3 profiling for MG3-1 and MG3-2 samples derived through Illumina MiSeq and phylogenetic distribution of OTUs as per direct QIIME analysis (QIIME round-1) of 16S rRNA V3 sequence data.

No.	Attribute	MG3-1	MG3-2	OTU distribution (%): Phylum level[#]		
				Phylum	MG3-1	MG3-2
1	DNA concentration (Qubit; ng µl^{-1})	0.31	1.28	Firmicutes	44.30	35.69
2	Total raw reads (Paired-end)	670,253	867,752	Proteobacteria	22.28	22.56
3	Sequence length	150	150	Bacteroidetes	14.43	14.81
4	Total Data (Mb)	201.07	260.32	Actinobacteria	8.61	15.15
5	%GC	54.66	54.75	Unknown	3.80	4.04
6	Phred score	35.42	35.21	Cyanobacteria	2.78	3.03
7	Read Phred quality ≥ Q30	90.45	89.60	TM7	1.27	1.01
8	Contig Length –Av	140	140	Fusobacteria	1.27	1.01
9	Reads passing conserved region filter	525,530	663,991	Acidobacteria	0.51	1.35
10	Reads passing Spacer	525,092	663,491	Nitrospirae	0.25	0.67
11	Reads passing read quality filter	525,039	663,428	Verrucomicrobia	0.25	0.34
12	Reads passing mismatch filter/ Consensus Reads	442,020	554,245	Tenericutes	0.25	-
13	Chimeric Sequences	560	439	Spirochaetes	-	0.34
14	Pre-processed Reads	441,460	553,806	**Further distribution of assignable OTUs**		
15	Total OTUs Picked	701	533	Class level	25†	26†
16	Total singleton OTUs	306	236	Order level	45†	42†
17	Total OTUs after singleton removal	395	297	Family level	75†	71†
				Genus	93†	81†
				Species	47†	41†

[#]based on 395 and 297 OTUs in MG3-1 and 3-2, respectively; †: Assignable taxonomic units excluding unknown OTUs

Table S3. Data on 16S rRNA metagene V3-V4 profiling for MG11 and MG12 samples derived through Illumina MiSeq and QIIME analysis of sequence data directly and after excluding chloroplast, mitochondrial and unassigned sequences showing the distribution of OTUs under different phyla.

No.	Attribute	**QIIME analysis-I** (including chloroplast, mitochondrial and unassigned sequences)		**QIIME analysis-II** (excluding chloroplast, mitochondrial and unassigned sequences)	
		**MG11†**	**MG12‡**	**MG11**	**MG12**
1	DNA concentration (ng μl^{-1})	2.4	4.1	2.4	4.1
2	Total Data (Mb)	346	599	130	238
3	No. of reads	755474	1321268	327531	605757
4	Mean seq. length	229.4	227	198.6	196.9
5	Min seq. length	40	40	40	40
6	Max seq. length	300	300	300	300
7	GC%	55.0	55.0	55.0	55.0
8	Stitch reads	430019	718373	2076	2862
9	Mean seq. length of stitch read	457.2	457.4	365.1	359.2
10	Min seq. length of stitch read	55	44	55	44
11	Max seq. length of stitch read	530	530	482	483
12	No. of reads after QC	429123	717185	1299	1670
13	No. of OTUs	558	720	455	581
14	Common OTUs	1277		1036	
15	α-diversity: Shannon index	0.869	0.848	7.846	8.041
16	Observed species	562	722	455	581
17	β-diversity	694123.1		233.60	

Distribution of OTUs under different phyla (values in percentage in decreasing order)

	**QIIME analysis -I**		**QIIME analysis –II**	
	**MG11**	**MG12**	**MG11**	**MG12**
1	Chloroplasts: 80.06	Chloroplasts: 81.98	Proteobacteria: 47.58	Proteobacteria: 42.69
2	Mitochondria: 18.99	Mitochondria: 17.13	Firmicutes: 15.47	Firmicutes: 19.46
3	Unassigned: 0.69	Unassigned: 0.72	Bacteroidetes: 12.78	Actinobacteria: 14.01
4	Proteobacteria: 0.14	Proteobacteria: 0.09	Actinobacteria: 11.78	Bacteroidetes: 10.90
5	Firmicutes: 0.04	Firmicutes: 0.04	Planctomycetes: 2.39	Planctomycetes: 3.35
6	Bacteroidetes: 0.04	Actinobacteria: 0.03	Cyanobacteria: 1.92	Cyanobacteria: 1.92
7	Actinobacteria: 0.03	Bacteroidetes: 0.01	Chlamydiae: 1.31	Chlamydiae: 0.18
8	Planctomycetes: 0.01		Verrucomicrobia: 1.08	Verrucomicrobia: 0.96
9			OD1: 1.08	OD1: 0.24

10		Chloroflexi: 1.08	Chloroflexi: 1.92
11		TM6: 0.77	TM6: 1.20
12		Fusobacteria: 0.77	Fusobacteria: 0.36
13		WPS-2: 0.38	Acidobacteria: 0.48
14		Acidobacteria: 0.38	TM7: 0.17
15		SC4: 0.23	Thermi: 0.18
16		TM7: 0.15	Nitrospirae: 0.24
17		Spirochaetes: 0.15	Chlorobi: 0.36
18		Thermi: 0.08	Tenericutes: 0.36
19		Nitrospirae: 0.08	SR1: 0.18
20		BRC1: 0.08	GN02: 0.12
21		**Euryarchaeota:** 0.46	Armatimonadetes: 0.12
22			Unassigned: 0.12
23			WS3: 0.06
24			OP8: 0.06
25			Gemmatimonadetes: 0.06
26			**Euryarchaeota 0.30**

†MG11 grape callus DNA sample: Mo Bio PowerFood® kit regular protocol

‡MG 12 grape callus DNA sample: Mo Bio PowerFood® kit extended protocol

Supplementary Figures

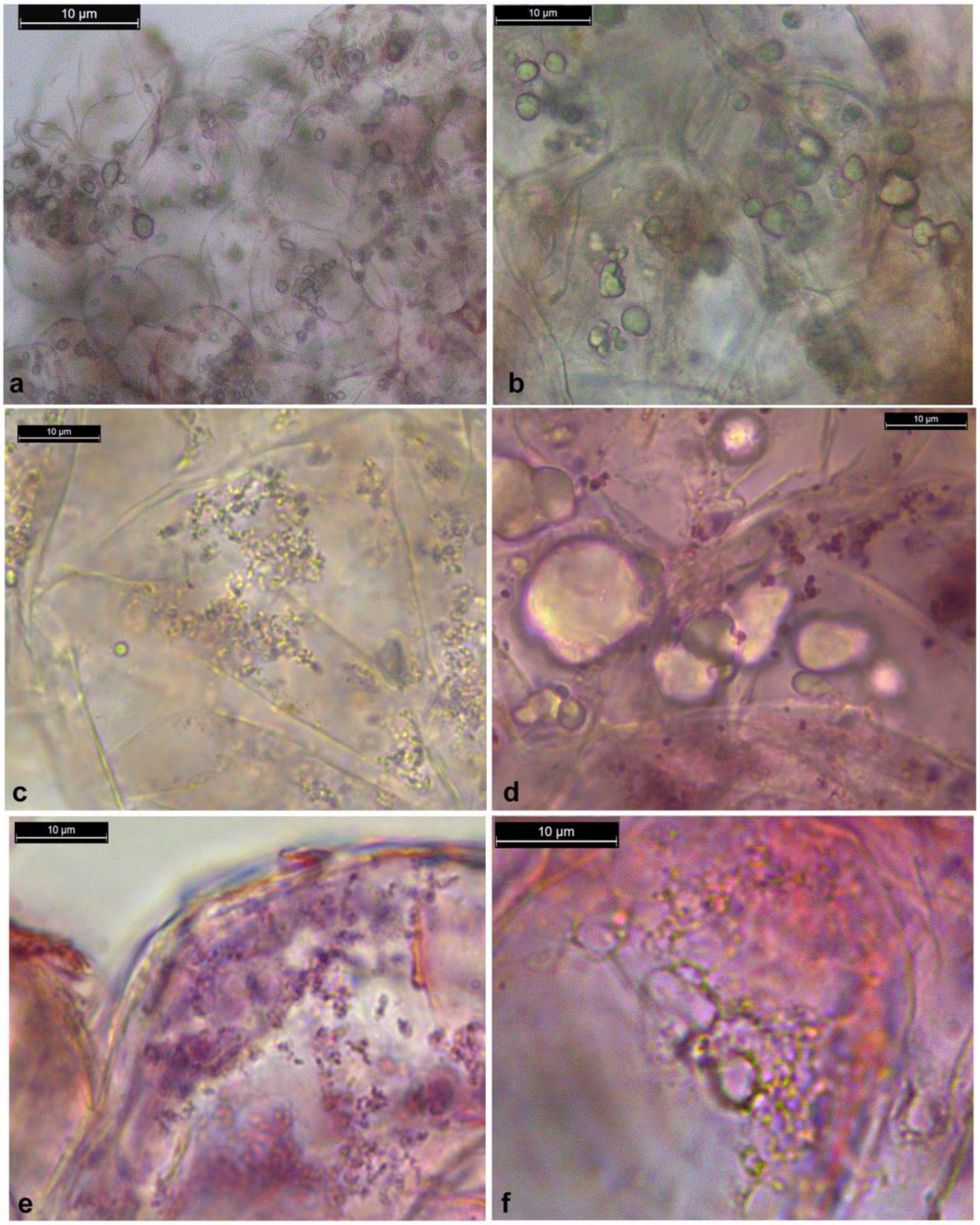

Figure S1. Bright-field microscopy on non-fixed 'FU01' grape suspension culture. (**a**) Cells under 100× magnification appearing in small clusters (20-40 μm) with red-pigmented patches under low magnification; (**b**) at 400× magnification displaying plastids and mitochondria as larger irregular or circular objects, (**c-f**) at 1000× magnification, (**c**) cells fixed in 4% formalin showing abundant grainy non-motile micro-particles that correspond to cellular bacteria, (**d, e**) formalin fixed cells stained with 0.005% safranin displaying pink stained bacterial cells with larger plastids and mitochondria, and (**f**) an intact grape cell stained with safranin displaying bacterial cells adhering to organelles (bar = 10 μm).

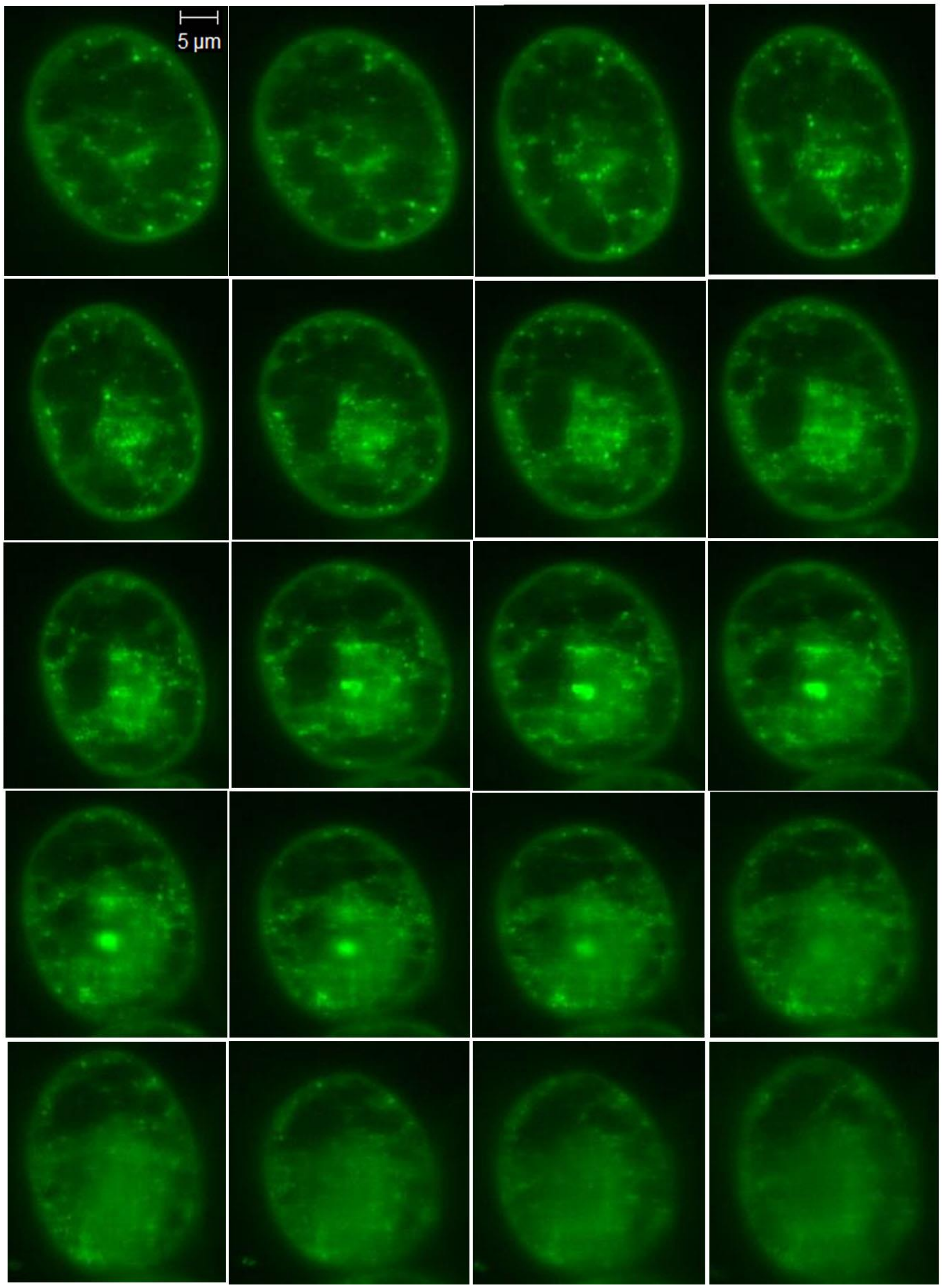

Figure S2. LSM5 live Confocal z-scan images (at 1 µm) on grape cell culture 'FU01'. Isolated grape cell derived after cellulase treatment stained with SYTO-9 displaying green fluorescing bacteria in the cytoplasm and along the perispace and adhering to the nucleus (bar = 5 µm).

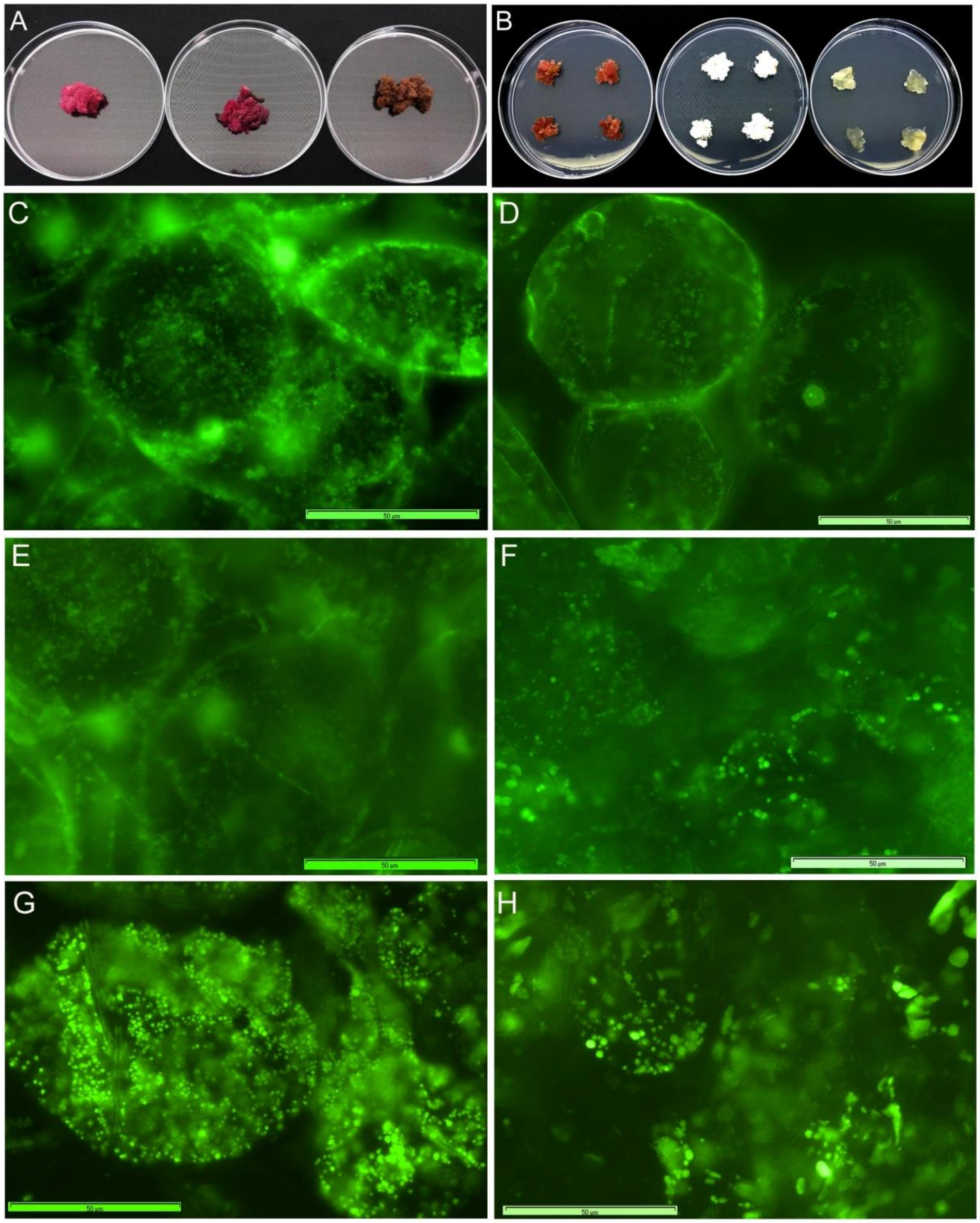

Figure S3. Callus stocks from different plant sources and laboratories in direct view or with epi-fluorescence microscopy after SYTO-9 staining. (**a**) Grape "FC01" callus on MS-agar-based callusing medium after one, two or four months of culturing (left to right), (**b**) one month-old callus cultures of "Gamay Freaux" "FC01", "Flame Seedless" and "Thompson Seedless" (left to right), (**c**) callus stock "FC-04" of grape "Pinot Noir", (**d**) "Chardonnay", and (**e**) "Cabernet Sauvignon", (**f**) callus stock of *Catharanthus roseus*, (**g**) callus stock of barley "Golden Promise" and (**h**) callus stock of barley "Sloop'" (Bar = 50 μm).

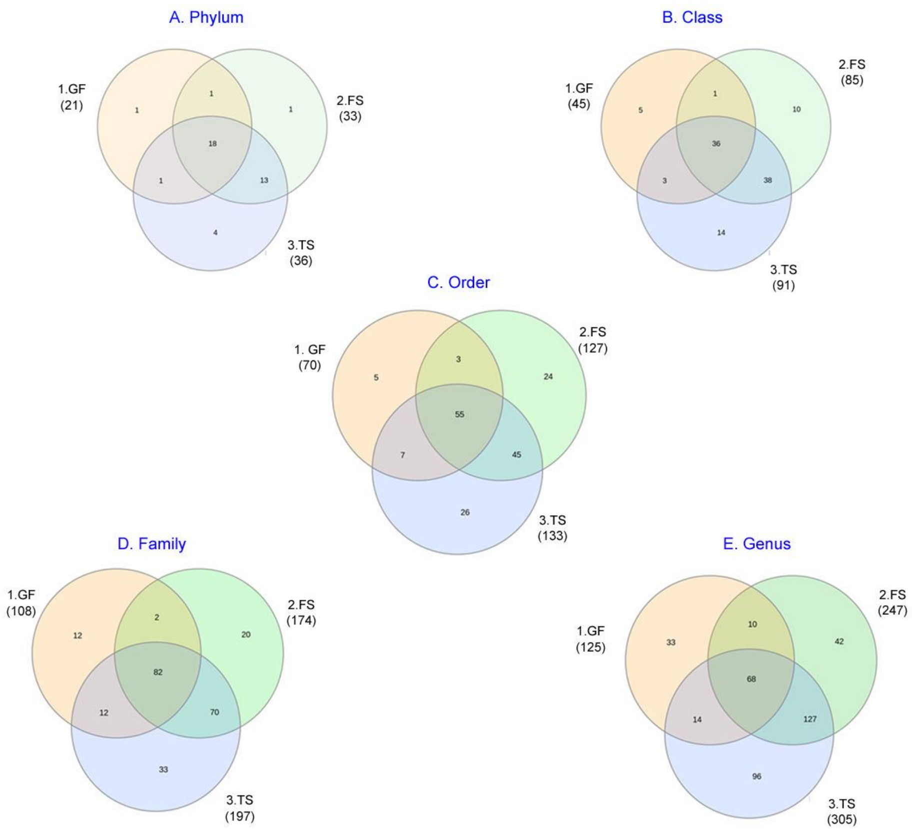

F. Defined genera common to three callus samples (40 nos; OTU share 14.01-.0.06%):
Acinetobacter,Lactobacillus, Propionibacterium,Corynebacterium, Aerococcus, Paracoccus, Micrococcus,Prevotella, Acetobacter, Pseudomonas, Staphylococcus, Clostridium, Kocuria, Streptococcus, [Prevotella], Rheinheimera, Oscillospira, Flavobacterium, Agrobacterium, Devosia, Jeotgalicoccus, Brachybacterium, Dechloromonas, Bacillus, Wautersiella, Porphyromonas, Haemophilus, Alloiococcus, Enhydrobacter, Neisseria, Sphingomonas, Kaistobacter, Fusobacterium, Alicyclobacillus, Lysobacter, Stenotrophomonas, Limnohabitans, Brevundimonas, Peptoniphilus, Dialister, Granulicatella, Macrococcus, Dietzia, Actinomyces

G. Defined genera unique to Gamay Freaux callus sample (70 nos; OTU share 1.32-0.06%)**:**
Desemzia, C39, Planktothricoides, Mycobacterium, Rhodobacter, Exiguobacterium, Flectobacillus, Sediminibacterium, Polynucleobacter, Ornithobacterium, CF231, Acidovorax, Methylocaldum, Synechococcus, Planktothrix, Arcobacter, Methylosinu, Megasphaera, Lactococcus, Deinococcus, Blvii28, Opitutus, Neptuniibacter, Nitrospira, Anaerolinea, Elizabethkingia, Prosthecobacter, Akkermansia, Geobacter, Desulfococcus, Rubrivivax, Comamonas, Anaerospora, Xanthobacter, Methylobacterium, Hyphomicrobium, Gemmata, Actinoplanes, Phascolarctobacterium, Dorea, Phycicoccus, Luteolibacter, Mycoplasma, Dokdonella, Photobacterium, Azomonas, Halomonas, Aggregatibacter, Serratia, Syntrophobacter, Georgfuchsia, Methylotenera, Ralstonia, Delftia, A17,Sutterella, Achromobacter, Pirellula, Pelosinus, Blautia, Proteiniclasticum, Leuconostoc, 5-7N15,Caldilinea, Emticicia, Adhaeribacter, Paludibacter, Dermacoccus, Dermabacter, Cellulomonas,Candidatus Solibacter

H. Defined genera unique to Flame Seedless callus sample (11 nos; OTU share 0.1%):
Odoribacter, YRC22, Mucispirillum, Symbiobacterium, Gallicola, Roseomonas, Alishewanella, Kushneria, Luteimonas, Thermomonas, Meiothermus

I. Defined genera unique to Flame Seedless callus sample (20 nos; OTU share 2.0-0.1%):
GW-34, 1-68, Arthrobacter, ph2, Veillonella, Marinobacter, Georgenia, Ornithinimicrobium, Yeosuana, Sphingobacterium, Geobacillus, Oribacterium, Balneimonas, Amaricoccus, Rubellimicrobium, Lautropia,Hydrogenophaga, Cellvibrio, Actinobacillus, Psychrobacter

Figure S4. VENN diagram showing the distribution of phylogenetic taxa in callus tissues of three grape cultivars “Gamay Freaux” (GF), “Flame Seedless” (FS) and “Thompson Seedless” (TS) at different taxonomic levels. (**a**) Common / distinct taxa defined at phylum, (**b**) class, (**c**) order, (**d**) family and (**e**) genus levels based on gross diversity; (**f**) List of common genera in the three callus stocks and (**g**) unique genera in “Gamay Freaux”, (**h**) “Flame Seedless” and (**i**) “Thompson Seedless” calluses; **f-i** based on defined genera only. [Comparison of defined genera present in the three callus tissue samples showed 44 common to all three samples; 6 genera common to “Flame Seedless” and “Flame Seedless”, 10 genera common to “Flame Seedless” and “Thompson Seedless”; 30 genera common to “Flame Seedless” and

“Thompson Seedless”. 70 genera appeared unique to “Gamay Freaux”, 11 unique to “Flame Seedless” and 20 genera unique to “Thompson Seedless”].
